# Sequential Double “Clicks” toward Structurally Well‐Defined Heterogeneous *N*‐Glycoclusters: The Importance of Cluster Heterogeneity on Pattern Recognition In Vivo

**DOI:** 10.1002/advs.201600394

**Published:** 2016-11-28

**Authors:** Liliya Latypova, Regina Sibgatullina, Akihiro Ogura, Katsumasa Fujiki, Alsu Khabibrakhmanova, Tsuyoshi Tahara, Satoshi Nozaki, Sayaka Urano, Kazuki Tsubokura, Hirotaka Onoe, Yasuyoshi Watanabe, Almira Kurbangalieva, Katsunori Tanaka

**Affiliations:** ^1^ Biofunctional Synthetic Chemistry LaboratoryRIKEN, HirosawaWako‐shi, Saitama351‐0198Japan; ^2^ Biofunctional Chemistry LaboratoryA. Butlerov Institute of ChemistryKazan Federal University18 Kremlyovskaya streetKazan420008Russia; ^3^ Center for Life Science TechnologiesRIKENMinatojima‐minamimachi, Chuo‐kuKobe, Hyogo650‐0047Japan; ^4^ JST‐PRESTO, HirosawaWako‐shi, Saitama351‐0198Japan

**Keywords:** biodistribution, heterogeneity, in vivo kinetics, *N*‐glycoalbumin, noninvasive fluorescence imaging, pattern recognition

## Abstract

**Structurally well‐defined heterogeneous *N*‐glycoclusters** are prepared on albumin via a double click procedure. The number of glycan molecules present, in addition to the spatial arrangement of glycans in the heterogeneous glycoclusters, plays an important role in the in vivo kinetics and organ‐selective accumulation through glycan pattern recognition mechanisms.

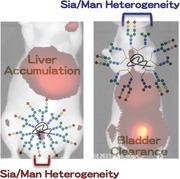

Living organisms use a diverse set of resources composed of simple and complex carbohydrates and glycoconjugates for their vital activities. Asparagine‐linked glycans (*N*‐glycans) and their glycoconjugates play particularly important roles in the innate immune response, adhesion, or receptor‐mediated signal transduction^1^ based on cell surface recognition through pattern recognition mechanisms.^2^, ^3^, ^4^, ^5^ Glycan multivalency and heterogeneity effects may be important parts of these mechanisms. Interestingly, a clear molecular basis for pattern recognition based on glycan‐specific multivalency and heterogeneous effects, e.g., on cell surfaces, has yet to be extensively explored.

Several types of glycoclusters use glycan multivalency effects to form selective interactions with target lectins.^6^, ^7^, ^8^, ^9^, ^10^, ^11^, ^12^ Recently, the unique and heterogeneous interactions between various glycans, lectins, and antibodies have been characterized using microarray technologies.^13^, ^14^ Interest has now shifted toward elucidating how glycan multivalency effects might control molecular kinetics in live animals. This work seeks to develop glycocluster‐based diagnostic and theranostic tracers that could be superior to existing peptide‐ or antibody‐based tracers with respect to selectivity and sensitivity. Glycocluster‐based dendrimers, liposomes, and nanoparticles have been examined using in vivo kinetics and/or biodistribution studies.^15^, ^16^, ^17^, ^18^, ^19^, ^20^, ^21^, ^22^, ^23^, ^24^, ^25^, ^26^, ^27^, ^28^, ^29^ In an effort to design multivalency into certain proteins of interest, several studies have attempted to immobilize monosaccharides or glycans onto proteins using bioorthogonal click reactions,^30^, ^31^, ^32^, ^33^ reactions around the amino group in lysine residues^25^, ^30^, ^34^, ^35^ or the thiol group in cysteine,^30^, ^36^, ^37^, ^38^ or using enzymatic glycosylation.^30^, ^39^, ^40^, ^41^

In studies of *N*‐glycan‐related compounds, Gabius and co‐workers demonstrated that immobilizing a few *N*‐glycans onto ^125^I‐labeled bovine serum albumin (BSA) moderately regulated the biodistributions and serum stabilities of the proteins in mice.^25^, ^26^, ^27^, ^42^ The presence of sialic acid residues in the glycoalbumin stabilized the molecules in serum, consistent with the receptor‐mediated excretion mechanism associated with asialoglycoprotein.^43^, ^44^, ^45^ The use of a double “click” methodology developed by our group enables the immobilization of a large number of glycans per albumin to facilitate glycan cluster‐dependent biodistribution and kinetics studies.^46^, ^47^ A strain‐promoted click reaction (alkyne–azide cycloaddition reaction)^48^ followed by the subsequent 6π‐azaelectrocyclization of unsaturated imines (RIKEN click reaction)^46^, ^47^, ^49^, ^50^, ^51^, ^52^, ^53^, ^54^ provides an efficient method for directly modifying native lysines and immobilizing up to a dozen complex‐type *N*‐glycans on albumin, resulting in the synthesis of homogeneous glycoalbumins, **Figure** **1**a. Noninvasive fluorescence images of these homogenous glycoalbumins in mice revealed that the *N*‐glycoalbumins accumulated in different organs (liver, spleen, or tumor), or were differentially excreted, depending on the *N*‐glycan structure. For example, α(2,6)‐sialoglycoalbumin **2a** was excreted through the urinary bladder, whereas galactosyl albumin **2b** was cleared through the gallbladder and then the intestine (Figure 1b). These effects could be explained in terms of two plausible excretion mechanisms operating in the liver parenchymal cells. Sialoglycoalbumin **2a** appeared to weakly and reversibly bind to the asialoglycoprotein receptor (ASGPR).^55^ Upon release, **2a** then appeared to be metabolized for excretion in the urinary bladder through biofiltration.^56^ On the other hand, galactosyl glycoalbumin **2b** could be recognized and endocytosed by ASGPR and then transported to the gallbladder and intestine via the polar transportation mechanism.^57^ As an example of accumulation of our glycoalbumins, the hybrid‐type glycocluster **2c** was selectively accumulated in the liver. **2c** contains different glycan arms linked to a branching mannose, i.e., α(2,6)‐sialic acid and mannose. Nonparenchymal cells, such as liver stellate cells, are largely responsible for liver‐specific accumulation (Figure 1b).^46^

**Figure 1:**
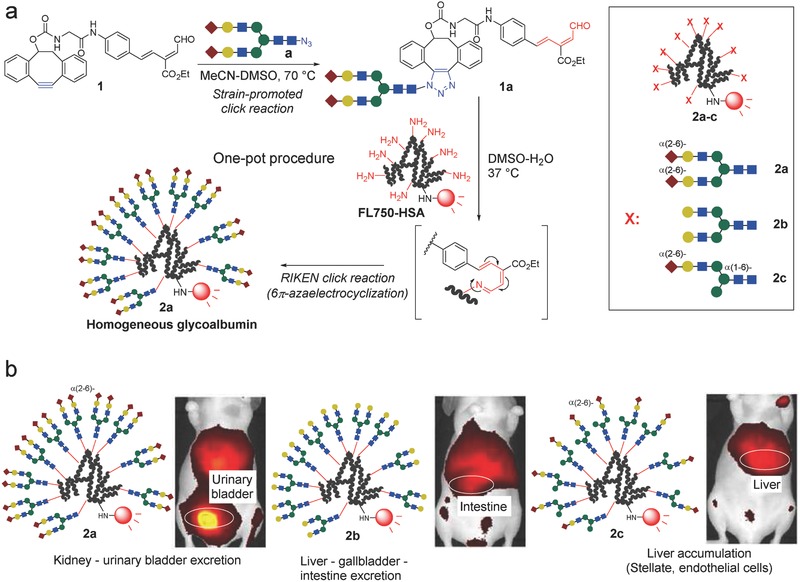
a) Preparation of homogeneous *N*‐glycoalbumins through a combination of a strain‐promoted click reaction and a RIKEN click reaction (6π‐azaelectrocyclization). b) Noninvasive fluorescence images of homogeneous glycoalbumins **2a**–**2c** in BALB/c nude mice 3 h after intravenous injection.

For comparison, heterogeneous glycoalbumins with different ratios of α(2,6)‐sialic acid and galactose‐terminated *N*‐glycans **2d**–**2f** were successfully synthesized (**Figure** **2**a). The use of the double click strategy outlined in Figure 1a allowed us to control the amounts of each glycan introduced onto albumin by adjusting the concentration of the RIKEN click probes **1a** and **1b**. An in vivo kinetics study revealed an almost linear correlation between the amounts of sialo‐ and galactose‐terminated glycans immobilized onto the albumin, **2d**–**2f**, and the excretion properties of these constructs, i.e., to the urinary bladder or the gallbladder/intestine (Figure 2b).^46^ These results presented the first examples of the effects of heterogeneity on in vivo kinetics.

**Figure 2:**
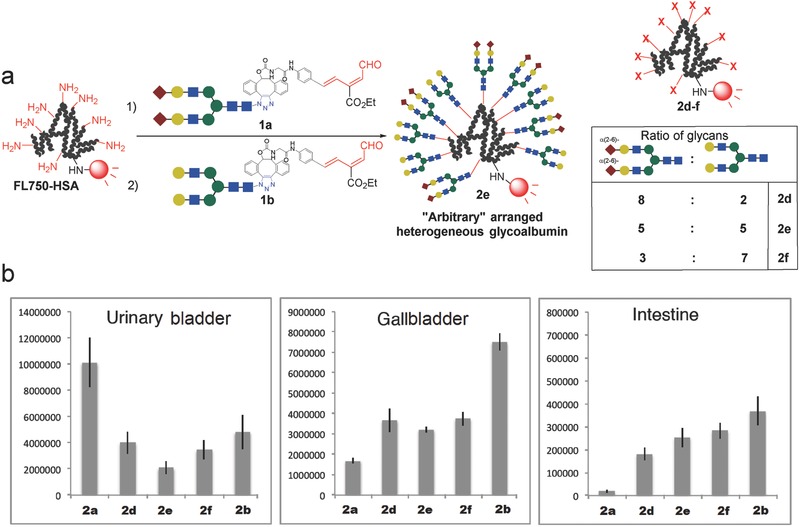
a) Preparation of arbitrarily arranged heterogeneous *N*‐glycoalbumins through a sequential RIKEN click reaction involving two glycan probes **1a** and **1b**. b) The fluorescence intensities of the excreted glycoalbumins **2a**, **2b**, **2d**–**2f** in the urinary bladder, gallbladder, and intestine obtained from BALB/c nude mice over 3 h after intravenous injection.

Although the heterogeneous glycoclusters **2d**–**2f** contained specific amounts of two different glycans in a controlled ratio, the positions of the glycans within the molecules and their arrangement relative to one another could not be precisely controlled (**Figure** **3**a). The glycoalbumins **2d**–**2f**, illustrated in Figure 2, could be described as “arbitrarily” arranged or “positionally” uncontrolled heterogeneous glycoalbumins. From this point of view, the syntheses and in vivo kinetic analyses of structurally well‐defined heterogeneous glycoclusters remains underexplored. One approach to addressing this problem using our double click strategy includes the initial incorporation of two distinct glycan moieties onto one azide unit, followed by linkage to albumin (Figure 3b). In this case, the locations of the two glycans relative to one another are preorganized and, therefore, the spatial arrangement of the two glycans reduces one aspect of heterogeneity among the resulting glycoalbumins. In vivo kinetics and biodistribution studies of structurally well‐defined heterogeneous *N*‐glycoalbumins could contribute significantly to an understanding of the importance of multivalency and heterogeneity effects during pattern recognition processes in mouse models.

**Figure 3:**
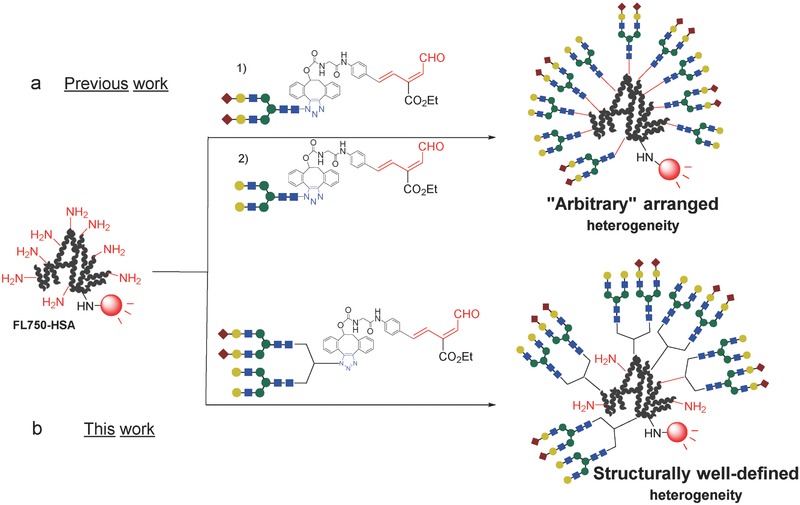
Synthesis of two types of heterogeneous *N‐*glycoalbumins using the RIKEN click strategy. a) Arbitrarily arranged heterogeneous glycoalbumins prepared previously. b) Structurally well‐defined heterogeneous glycoalbumins reported in this paper.

In this paper, we describe the synthesis of structurally well‐defined heterogeneous *N*‐glycoclusters containing (i) Sia/Gal‐terminated and (ii) Sia/Man‐terminated *N*‐glycans. We compared their excretion and accumulation properties with those of glycoalbumins prepared using existing methods. This research uncovered notable differences among three types of glycoalbumins: homogeneous glycoalbumins, arbitrarily arranged heterogeneous glycoalbumins, and structurally well‐defined heterogeneous glycoalbumins; thereby demonstrating, for the first time, the importance of multivalency and heterogeneity in pattern recognition in vivo.

The synthesis of structurally well‐defined heterogeneous glycoalbumins was envisioned using a new strategy, as depicted in Figure 3b, namely, the incorporation of two different *N*‐glycans into a single azide unit, and the subsequent clusterization onto a near‐infrared fluorescence (HiLyte Fluor 750)‐labeled albumin. Initially, α(2,6)‐sialic acid and galactose‐terminated complex type *N*‐glycans were selected for incorporation into a unit (**Scheme** **1**). The excretion trends of these structurally well‐defined heterogeneous glycoclusters (**8a** in Scheme 1) were expected to differ from the excretion trends of the previously studied homogeneous (**2a**, **2b**) and arbitrarily arranged heterogeneous clusters (**2e**, see the excretion data in Figure 2b). The combination of α(2,6)‐sialic acid and mannose‐terminated *N*‐glycans in our new heterogeneous glycoalbumins (**9a** in Scheme 1) was also hypothesized to provide novel in vivo behaviors relative to the homogeneous glycoalbumin prepared with a hybrid‐type *N*‐glycan (**2c**, Figure 1b), which exclusively accumulated in the liver. The presence of numerous nonreducing terminal sialic acid and mannose moieties on the *N*‐glycans on the surface of glycoalbumin **9a** resembled the glycan structure found on **2c**, although the spatial arrangements among neighboring *N*‐glycans differed. We envisioned that bis‐succinimidyl ester/azide **3** might be an appropriate precursor for uniting two different *N*‐glycans and immobilizing them as a single heterogeneous unit on the albumin protein.

**Scheme 1:**
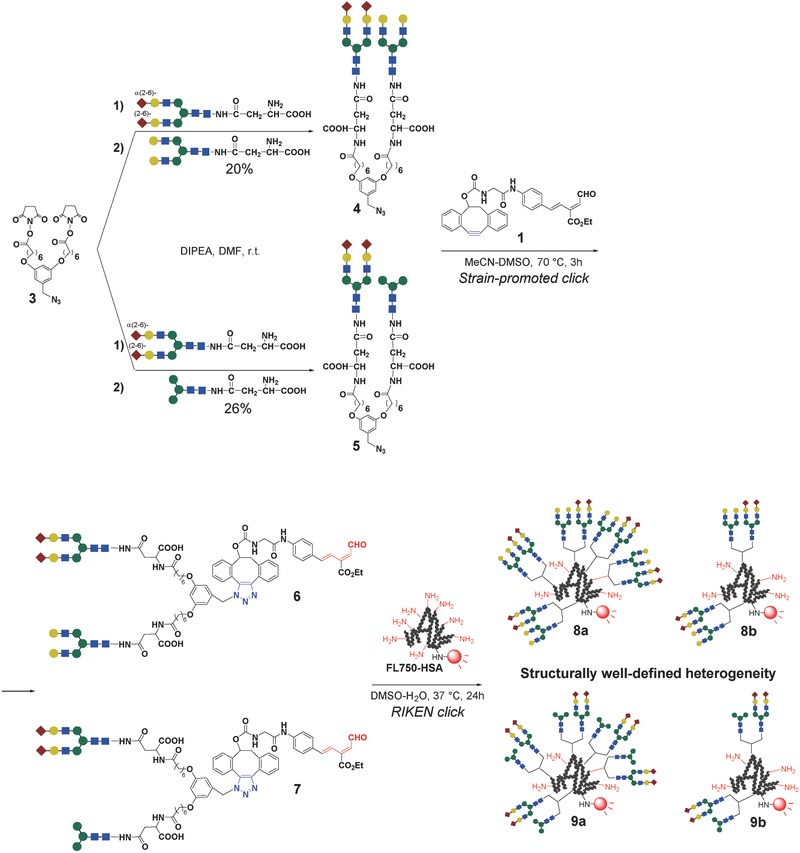
Synthesis of the azide units and subsequent immobilization on fluorescently labeled albumin (FL750‐HSA) via strain‐promoted click and RIKEN click reactions.

The reactions of bis‐succinimidyl ester **3** with two different *N*‐glycans in the presence of diisopropylethyl amine (DIPEA) in dimethylformamide (DMF) at room temperature produced the desired azides **4** and **5**, accompanied by the formation of the bis‐homo‐ and monosubstituted products (the details are described in the Supporting Information). It should be noted that the reactivity of the α(2,6)‐sialic acid‐terminated glycan was lower than the reactivities of the galactose‐ and mannose‐terminated glycans; hence, sequential treatment of the two glycans in a one‐pot procedure was essential for increasing the yields of the target azide units. The optimal reaction conditions were found to include initial treatment of compound **3** with sialoglycan over 4 h, followed by treatment with asialoglycan over 20 h in the same flask. The reaction mixtures were directly purified by high performance liquid chromatography (HPLC), and compounds **4** and **5** were obtained in 20% and 26% yields, respectively (see the Supporting Information for details).

The azides **4** and **5** were then immobilized onto fluorescently labeled albumin through a double click procedure involving a strain‐promoted click reaction, followed by the RIKEN click reaction, as developed previously.^46^ Although these azides contained two molecules of large complex *N*‐glycans, the RIKEN click reaction using the probes **6** and **7** allowed for the efficient immobilization of even five azide units (for a total of ten *N*‐glycan molecules) per albumin, as determined by matrix‐assisted laser desorption/ionization time‐of‐flight (MALDI‐TOF) mass spectrometry (glycoalbumins **8a** and **9a**, see the Supporting Information for details). Intact or partially glycosylated albumins could not be observed in the mass spectra of the reaction mixtures; hence, the double click reaction was nearly quantitative with respect to the albumin concentration. These results demonstrated the efficient construction of glycoclusters on protein templates using our RIKEN click reaction. It is important to note that smaller numbers of glycan units could be immobilized on albumin by controlling the concentrations of the RIKEN click probes **6** and **7**. Thus, the heterogeneous **8b** and **9b**, with two units (for a total of four *N*‐glycan molecules) per albumin were prepared in a highly efficient manner (Scheme 1).

Noninvasive fluorescence imaging techniques were used to determine the excretion properties of the structurally well‐defined heterogeneous glycoalbumins **8a**, **8b** and **9a**, **9b**, and these results were compared with those reported previously using homogeneous and arbitrarily arranged heterogeneous glycoalbumins. As mentioned above, the homogeneous α(2,6)‐sialylated glycoalbumin **2a** was preferentially excreted through the urinary bladder, whereas the galactosylated glycoalbumin **2b** was excreted through the gallbladder and intestine (Figures 2b and 4d).^46^ Furthermore, positionally uncontrolled heterogeneous glycoalbumins containing both glycans (**2e**, with a Sia/Gal glycan ratio of 1:1) displayed excretion properties that were intermediate between those of the homogeneous clusters **2a** and **2b** (**Figure** **4**d). The structurally well‐defined heterogeneous glycoalbumin **8a** similarly exhibited excretion properties intermediate between those of the homogeneous **2a** and **2b** across the urinary bladder and gallbladder excretion pathways (Figure 4a,d). On the other hand, the intestine displayed a much higher fluorescence intensity in mice dosed with the structurally well‐defined heterogeneous glycoalbumin **8a** than in mice dosed with **2e** or even galactosyl glycoalbumin **2b**. The immobilization of two identical sialoglycan and asialoglycan units on the azide **3** and the subsequent clustering on albumins resulted in excretion properties identical to those of **2a** and **2b** (data not shown). These results suggested that the structurally defined glycoalbumin **8a** could be very rapidly translocalized from the gallbladder to the intestine for excretion. The structurally well‐defined heterogeneous **8b**, which contained fewer glycan molecules, namely four glycans per albumin, was also excreted very rapidly to the intestine, similar to the glycoalbumin **8a**, which was characterized by a higher glycan valency (Figure 4b–d). Thus, even small amounts of glycans immobilized onto albumin were sufficient to produce multivalency and heterogeneity effects in the gallbladder/intestine translocalization properties in the context of structurally well‐defined heterogeneous cluster environments.

**Figure 4:**
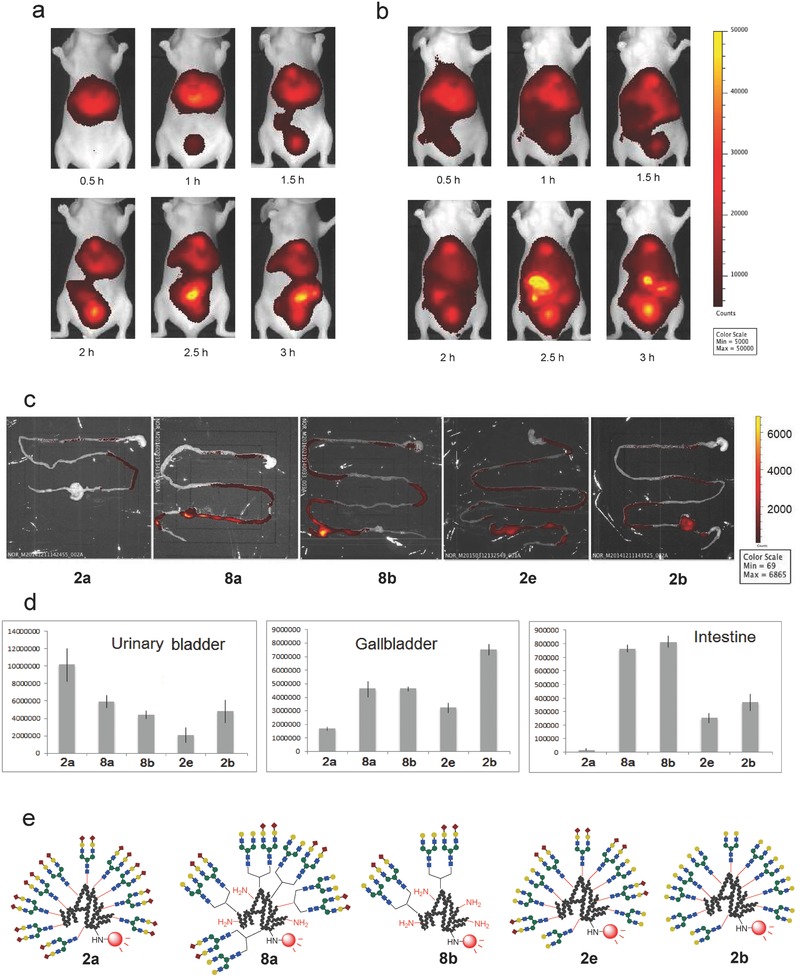
Noninvasive fluorescence imaging of the *N*‐glycoclusters a) **8a** and b) **8b** in BALB/c nude mice 0.5–3 h after intravenous injection. c) Fluorescence intensities of the dissected intestines 3 h after administration of the glycoalbumins **2a**, **8a**, **8b**, **2b**, and **2e**. d) Comparison of the fluorescence intensities of the excreted glycoalbumins **2a**, **8a**, **8b**, **2b**, and **2e** in the urinary bladder, gallbladder, and intestine, from mice over 3 h after injection. See the procedural details provided in the Experimental Section. e) Structures of the glycoalbumins.

Previous studies showed that the hybrid‐type homogeneous glycoalbumin **2c**, which contains both α(2,6)‐sialic acid and mannose‐terminated branches on the glycan structure, was strongly and selectively accumulated in the liver (Figure 1b).^46^ Noninvasive fluorescence imaging of the structurally well‐defined heterogeneous *N*‐glycoalbumins **9a** and **9b**, however, revealed in vivo kinetics that were quite unique and entirely distinct from those of **2c**. The excretion and liver accumulation properties depended on the glycan multivalency on albumin; thus, glycoalbumin **9a**, containing a total of ten glycan molecules per albumin, was excreted smoothly through the urinary bladder (**Figure** **5**a,d). Glycocluster **9b**, containing fewer glycans on albumin, on the other hand, preferentially accumulated in the liver (Figure 5b–d).

**Figure 5:**
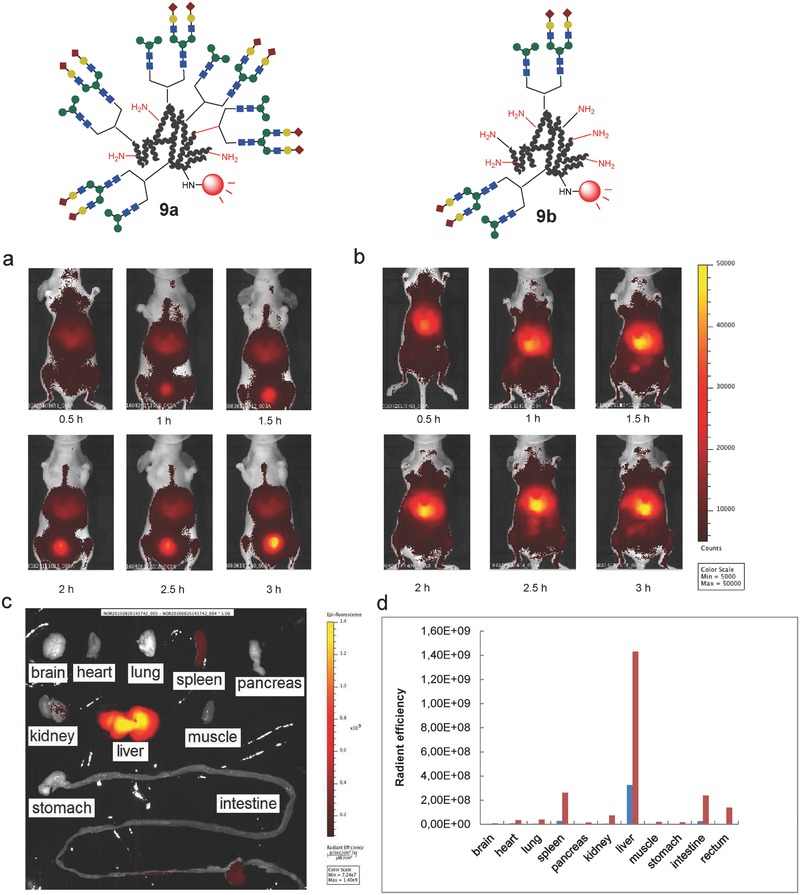
Noninvasive fluorescence images of the *N*‐glycoclusters a) **9a** and b) **9b** in BALB/c nude mice 0.5–3 h after intravenous injection. c) Fluorescence intensities of the dissected organs 3 h after administration of glycoalbumin **9b**. d) Comparison of the fluorescence intensities of the dissected organs 3 h after administration of **9a** and **9b**. Blue: **9a**, Red: **9b**. See the procedural details provided in the Experimental Section.

The message gleaned from in vivo data collected using three types of glycoalbumins, namely, (i) homogeneous, (ii) arbitrarily arranged heterogeneous, and (iii) structurally well‐defined heterogeneous glycoalbumins, was that the spatial arrangements of *N*‐glycans within heterogeneous glycoclusters, in addition to the number of glycan molecules immobilized on albumin, played an important role in the in vivo kinetics and organ‐selective accumulation through glycan pattern recognition mechanisms.

In summary, a synthetic strategy for preparing structurally well‐defined heterogeneous glycoclusters was established. The incorporation of two distinct glycans into a single azide unit, followed by efficient clusterizing on albumin using a double click strategy, permitted us to control the spatial arrangement of the two glycans on albumin. In vivo imaging and biodistribution studies revealed, for the first time, the importance of “glycan heterogeneity in vivo.” The properties of the constructs developed here were compared with those of previously prepared homogeneous and arbitrarily arranged heterogeneous congeners. Thus, structurally well‐defined α(2,6)‐Sia/Gal‐terminated heterogeneous glycoalbumins exhibited nonidentical excretion properties that depended on the arrangement heterogeneity of the glycans, resulting in rapid translocalization from the gallbladder to the intestine, even when compared with Gal‐terminated clusters. The structurally well‐defined α(2,6)‐Sia/Man‐terminated heterogeneous glycoalbumins were structurally distinct from the hybrid‐type glycoalbumins, and their in vivo behavior was quite unique. These constructs were selectively excreted through the urinary bladder, or they accumulated in the liver, depending on the albumin‐bound glycan valency. These data clearly indicated that the dimensional arrangement of glycans relative to one another within the heterogeneous glycoclusters was very important for enabling pattern recognition in vivo. In other words, if used efficiently, heterogeneity‐dependent pattern recognition may provide precise control over the excretion or organ‐selective accumulation of molecules of interest in live organisms, leading to new strategies for the development of glycan‐based imaging or therapeutic tracers. Tracers with these properties cannot be prepared using known molecules. An exploration of the various heterogeneous glycoclusters that are either structurally well‐defined or having heterogeneity through the arbitrary arrangement of glycans may lead to targeting specific disease organs in animal models, e.g., specific tumors. This work is in progress in our laboratory.

## Experimental Section

5‐(Bromomethyl)benzene‐1,3‐diol, 5‐(azidomethyl)benzene‐1,3‐diol, and cyclooctyne‐aldehyde were synthesized according to procedures reported in the literature.^50^, ^58^ 3,5‐Dihydroxybenzyl alcohol, carbon tetrabromide, triphenylphosphine, sodium azide, and *N*‐hydroxysuccinimide (NHS) were purchased from Acros, ethyl 7‐bromoheptanoate and human serum albumin (HSA) were obtained from Sigma‐Aldrich, 1‐ethyl‐3‐(3‐(dimethylamino)propyl)carbodiimide hydrochloride (EDC) was provided by TCI, *N*‐glycans were supplied from Glytech, Inc., and the fluorescent compound HiLyte Fluor750 acid SE was provided by AnaSpec, Inc. Fremont, USA. All other commercially available reagents were used without further purification.

The syntheses of diethyl 7,7′‐((5‐azidomethyl)‐1,3‐phenylene)bis(oxy)diheptanoate, 7,7′‐((5‐azidomethyl)‐1,3‐phenylene)bis(oxy)diheptanoic acid, di‐*N*‐hydroxysuccinimide 7,7′‐((5‐azidomethyl)‐1,3‐phenylene)bis(oxy)diheptanoate **3**, the Sia/Gal‐terminated azide unit **4**, and the Sia/Man‐terminated azide unit **5** are described in the Supporting Information.

*Synthesis of the Structurally Well‐Defined Heterogeneous Glycoalbumins*: The azides **4** and **5** (160 × 10^−9^ m) were initially treated with freshly prepared cyclooctyne–aldehyde (150 × 10^−9^ m). Heating the mixture hastened the quantitative completion of the strain‐promoted click reaction (the production of the RIKEN click probes **6** and **7**), which could be conveniently monitored by HPLC. An aqueous solution of the HiLyte Fluor 750‐labeled HSA^46^ (9.7 × 10^−9^ m) was added to this mixture, and the RIKEN click reaction was proceeded with heating at 37 °C to ensure conjugation of the glycan units to albumin. After small molecules were filtered off using an Amicon centrifugal filter, *N*‐glycan conjugation was evaluated by MALDI‐TOF mass spectroscopy. By controlling the concentrations of the RIKEN click probes **6** and **7**, approximately five or two azide units, hence, a total of ten or four glycan molecules, could be introduced onto each albumin (glycoalbumins **8a**, **8b** and **9a**, **9b**). The analytical results are described in detail in the Supporting Information.

*Excretion and Biodistribution Analysis of the Glycoalbumins in BALB/c Nude Mice*: 1.5 nmol per 30 µL of the *N*‐glycoalbumins **8a**, **8b** and **9a**, **9b** labeled with the near‐infrared fluorescent probe (HiLyte Fluor 750) were diluted in 70 µL saline and injected into eight to ten week‐old BALB/cAJcl‐nu/nu mice via the tail vein (*N* = 4). The mice were then anesthetized with pentobarbital or isoflurane and placed in a fluorescence imager, IVIS kinetics fluorescence imager (Caliper Life Sciences, Inc., Hopkinton, Massachusetts, USA). Abdominal and dorsal side images were collected at 30 min intervals. Although the fluorescence signals from the dorsal side did not exhibit significant differences in the trafficking and/or accumulation of the *N‐*glycoalbumins investigated (i.e., the compounds appeared evenly distributed across the whole body), signals from the abdominal side clearly revealed time‐dependent excretion as well as accumulation in specific organs. As a result, the abdominal data were used to draw conclusions from the in vivo kinetics and distribution studies. The fluorescence signal increased rapidly in the urinary bladder, and the fluorescence around the urinary bladder was calculated within an arbitrarily defined region of interest (ROI). After 3 h of observation, the mice were sacrificed and perfused with 4% paraformaldehyde solution, and the fluorescence intensities in the brain, heart, lung, spleen, pancreas, kidney, liver, muscle, stomach, intestine, and rectum were measured within the arbitrarily defined ROI.

All procedures involving experiment animals were approved by the Ethics Committee of RIKEN (MAH21‐19‐17). The experiments were performed in accordance with the institutional and national guidelines.

## Acknowledgements

L.L. and R.S. contributed equally to this work. This work was supported by the JSPS KAKENHI Grant Numbers JP16H03287, JP16K13104, and JP15H05843 in Middle Molecular Strategy. This work was also performed with the support of the Russian Government Program for Competitive Growth, granted to the Kazan Federal University. The authors thank Glytech, Inc. for supplying various *N*‐glycans.

## Supporting information

As a service to our authors and readers, this journal provides supporting information supplied by the authors. Such materials are peer reviewed and may be re‐organized for online delivery, but are not copy‐edited or typeset. Technical support issues arising from supporting information (other than missing files) should be addressed to the authors.

Supplementary
