# Supplementary material for: Sequential Double “Clicks” toward Structurally Well‐Defined Heterogeneous N‐Glycoclusters: The Importance of Cluster Heterogeneity on Pattern Recognition In Vivo
Source: Adv Sci (Weinh). 2016 Nov 28;4(2):1600394. doi: 10.1002/advs.201600394 (PMC5323863; doi:10.1002/advs.201600394)
Supplement: Supplementary file 1 — Supplementary [file ADVS-4-na-s001.pdf]

Copyright WILEY-VCH Verlag GmbH & Co. KGaA, 69469 Weinheim, Germany, 2016.

ADVANCED SCIENCE
Open Access

Supporting Information

for *Adv. Sci.,* DOI: 10.1002/advs.201600394

Sequential Double "Clicks" toward Structurally Well-Defined Heterogeneous *N*-Glycoclusters: The Importance of Cluster Heterogeneity on Pattern Recognition In Vivo

Liliya Latypova, Regina Sibgatullina, Akihiro Ogura, Katsumasa Fujiki, Alsu Khabibrakhmanova, Tsuyoshi Tahara, Satoshi Nozaki, Sayaka Urano, Kazuki Tsubokura, Hirotaka Onoe, Yasuyoshi Watanabe, Almira Kurbangalieva, and Katsunori Tanaka**

Copyright WILEY-VCH Verlag GmbH & Co. KGaA, 69469 Weinheim, Germany, 2013.

Supporting Information

Sequential Double "Clicks" Toward Structurally Well-defined Heterogeneous *N*-Glycoclusters: The Importance of Cluster Heterogeneity on Pattern Recognition *In Vivo*

Liliya Latypova, Regina Sibgatullina, Akihiro Ogura, Katsumasa Fujiki, Alsu Khabibrakhmanova, Tsuyoshi Tahara, Satoshi Nozaki, Sayaka Urano, Kazuki Tsubokura, Hirotaka Onoe, Yasuyoshi Watanabe, Almira Kurbangalieva and Katsunori Tanaka**

Materials. 3,5-Dihydroxybenzyl alcohol, carbon tetrabromide, triphenylphosphine, sodium azide, *N*-hydroxysuccinimide (NHS) were purchased from Acros, ethyl 7-bromoheptanoate, human serum albumin (HSA) were obtained from Sigma-Aldrich, 1-ethyl-3-(3-(dimethylamino)propyl)carbodiimide hydrochloride (EDC) was provided by TCI, *N*-glycans were supplied from Glytech, Inc., fluorescent compound HiLyte^{TM} Fluor750 acid SE® was provided by AnaSpec, Inc. Fremont. Ultrapure water from Merck Milli-Q Advantage® was used for all synthetic experiments described in this paper.

High-resolution mass spectra (HRMS) were obtained on Bruker micrOTOF-QIII spectrometer® by electron spray ionization (ESI-TOF-MS). Mass spectra of the glycan-conjugated albumins were obtained on Bruker autoflex spectrometer® by matrix assisted laser desorption ionization (MALDI-TOF MS), using 2,5-dihydroxybenzoic acid as matrix. IR spectra of solid compounds were recorded on a Bruker Tensor-27 spectrometer from samples dispersed in Nujol and placed between KBr plates. ^{1}H and $^{13}C\{^{1}H\}$ NMR spectra were measured on Bruker Avance III 400 spectrometers (400.17 MHz for ^{1}H and 100.62 MHz for $^{13}C\{^{1}H\}$) and JEOL RESONANCE AL400 (395.75 MHz for ^{1}H) in $CDCl_3$, acetone-d_6, D_2O at room temperature. The peak of the residual protonated solvent was used as the internal standard. Multiplicities are indicated as: s (singlet), bs (broad singlet), d (doublet), t (triplet), q

(quartet), quint (quintet), m (multiplet). Analytical thin layer chromatography (TLC) was carried out with silica gel plates «Merck» (silica gel 60, F254, supported on aluminium), using UV light as the visualizing agent. Column chromatography was performed on Silica gel 60 (Acros, 0.060–0.200 mm). Reverse and normal phase HPLC analysis/purification was performed on Shimadzu Prominence® system equipped with Nacalai tesque column (5C18-AR-300, 4.6 x 250 mm) and Hilic-phase Inertsil Hilic column (Amide, 4.6 x 150 mm) respectively. Two solvent systems, namely, A: H_2O containing 0.1% TFA and B: MeCN containing 0.1% TFA, were applied. The melting points were measured on an *OptiMelt Stanford Research Systems MPA100* automated melting point apparatus and were not corrected.

Synthesis of bis-succinimidyl ester/azide 3. The synthetic route includes 5 steps from the commercially available 3,5-dihydroxybenzyl alcohol **10** (**Scheme S1**). 5-(Bromomethyl)benzene-1,3-diol (**11**) and 5-(azidomethyl)benzene-1,3-diol (**12**) were synthesized according to the literature.[1]

Synthesis of diethyl 7,7'-((5-azidomethyl)-1,3-phenylene)bis(oxy)diheptanoate 13 (Scheme S1). Ethyl 7-bromoheptanoate (1.1 mL, 5.63 mmol) and K_2CO_3 (0.78 g, 5.63 mmol) were added to solution of azide **12** (0.31 g, 1.88 mmol) in DMF (15 mL). The reaction mixture was stirred for 17 hours at 80 °C under argon atmosphere. The mixture was cooled down to r.t. and then treated with 5% aqueous solution of citric acid (20 ml) at 0 °C. The resulting solution was extracted with ethyl acetate (2 x 20 mL). The combined organic layers were washed with water (3 x 10 mL), brine (30 mL) and dried under $MgSO_4$. The solvent was removed in vacuum to give product **13** as yellow oil. Yield: 0.84 g (94%), R_f 0.68 (EtOAc/dichloromethane, 1/7). IR (film), ν, cm^{-1}: 2979, 2939, 2863 (CH), 2100 (N_3), 1734 (C=O), 1597 (C=C). ^{1}H NMR (400 MHz, $CDCl_3$, $CHCl_3$ = δ 7.26), δ, ppm: 1.23 (t, 6H, ^3J = 7.1 Hz, CH_3), 1.29 – 1.51 (m, 8H, CH_2), 1.64 (quint, 4H, ^3J = 7.5 Hz, $\underline{CH_2}CH_2C(O)$), 1.76

(quint, 4H, ^3J = 6.5 Hz, $OCH_2\underline{CH_2}$), 2.29 (t, 4H, ^3J = 7.5 Hz, $\underline{CH_2}C(O)$), 3.91 (t, 4H, ^3J = 6.5 Hz, $O\underline{CH_2}$), 4.10 (q, 4H, ^3J = 7.1 Hz, $O\underline{CH_2}CH_3$), 4.22 (s, 2H, CH_2N_3), 6.38 (t, 1H, ^4J = 2.1 Hz, ArH), 6.40 (d, 2H, ^4J = 2.1 Hz, ArH) (**Figure S1a**). $^{13}C\{^1H\}$ NMR (100 MHz, $CDCl_3$, $CDCl_3$ = δ 77.0), δ, ppm: 14.2 (CH_3), 24.9 ($\underline{CH_2}CH_2C(O)$), 25.7, 28.8 (CH_2), 29.0 ($OCH_2\underline{CH_2}$), 34.3 ($\underline{CH_2}C(O)$), 54.9 (CH_2N_3), 60.2 ($O\underline{CH_2}CH_3$), 67.9 ($O\underline{CH_2}CH_2$), 101.0 (C_{arom}), 106.5 ($2C_{arom}$), 137.4 ($\underline{C_{arom}}$–CH_2N_3), 160.5 ($2\underline{C_{arom}}$–O), 173.7 (2C=O) (**Figure S1b**).

Synthesis of 7,7'-((5-azidomethyl)-1,3-phenylene)bis(oxy)diheptanoic acid 14 (Scheme S1). Solution of NaOH (0.23 g, 5.75 mmol) in water (5 mL) was added to solution of azide **13** (0.69 g, 1.44 mmol) in ethanol (35 mL). Reaction mixture was stirred at 40 °C for 9 hours and the solvents were removed under vacuum. The solid residue was dissolved in water (20 mL) and acidified with 5 M aq. HCl to pH 2. The solution was stirred at room temperature for 3 hours, extracted with ethyl acetate (2 x 25 mL) and then with trichloromethane (20 mL). The combined organic layers were dried under $MgSO_4$ and the solvent was evaporated to give yellow oil. Crystallization from tetrachloromethane yielded product **14** as colorless crystalline compound. Yield: 0.48 g (80%), R_f 0.44 (acetone/toluene, 1/2), m.p.: 71–72 °C. IR, ν, cm^{-1}: 3300 – 2600 broad (OH), 2118, 2094 (N_3), 1709 (C=O), 1606 (C=C). ^1H NMR (400 MHz, $CDCl_3$, $CHCl_3$ = δ 7.26), δ, ppm: 1.37 – 1.56 (m, 8H, CH_2), 1.68 (quint, 4H, ^3J = 7.3 Hz, $\underline{CH_2}CH_2C(O)$), 1.79 (quint, 4H, ^3J = 6.6 Hz, $OCH_2\underline{CH_2}$), 2.38 (t, 4H, ^3J = 7.3 Hz, $\underline{CH_2}C(O)$), 3.94 (t, 4H, ^3J = 6.6 Hz, $O\underline{CH_2}$), 4.24 (s, 2H, CH_2N_3), 6.40 (t, 1H, ^4J = 2.2 Hz, ArH), 6.43 (d, 2H, ^4J = 2.2 Hz, ArH) (**Figure S2a**). $^{13}C\{^1H\}$ NMR (100 MHz, $CDCl_3$, $CDCl_3$ = δ 77.0), δ, ppm: 24.5 ($\underline{CH_2}CH_2C(O)$), 25.6, 28.5 (CH_2), 28.7 ($OCH_2\underline{CH_2}$), 33.8 ($\underline{CH_2}C(O)$), 54.9 (CH_2N_3), 67.7 (OCH_2), 100.7 (C_{arom}), 106.7 ($2C_{arom}$), 137.43 ($\underline{C_{arom}}$–CH_2N_3), 160.6 ($2\underline{C_{arom}}$–O), 179.9 (2C=O) (**Figure S2b**).

Synthesis of di-*N*-hydroxysuccinimide 7,7'-((5-azidomethyl)-1,3-phenylene)bis(oxy)diheptanoate 3 (Scheme S1). *N*-hydroxysuccinimide (0.24 g, 2.09 mmol) and EDC hydrochloride (0.40 g, 2.09 mmol) were added to solution of compound **14** (0.38 g, 0.90 mmol) in DMF (15 mL) at 0 °C. Reaction mixture was stirred at room temperature for 22 hours under argon atmosphere. After the completion of the reaction, the mixture was cooled, washed with water (25 mL) and extracted with ethyl acetate (2 x 40 mL). The combined organic layers were dried under $MgSO_4$ and the solvent was evaporated. Resulting yellow oil was purified by column chromatography (eluent ethanol/dichloromethane, 1/20) to give product **3** as a colorless solid. Yield: 0.45 g (81%), R_f 0.86 (ethanol/dichloromethane, 1/20), m.p.: 54 °C. IR, ν, cm^{-1}: 2102 (N_3), 1813, 1784, 1741 (C=O), 1596 (C=C). ^1H NMR (400 MHz, $CDCl_3$, $CHCl_3$ = δ 7.26), δ, ppm: 1.41 – 1.57 (m, 8H, CH_2), 1.69 – 1.86 (m, 8H, CH_2), 2.62 (t, 4H, ^3J = 7.4 Hz, $\underline{CH_2}C(O)$), 2.82 (bs, 8H, $CH_2C(O)N$), 3.94 (t, 4H, ^3J = 6.4 Hz, $O\underline{CH_2}$), 4.24 (s, 2H, CH_2N_3), 6.40 (t, 1H, ^4J = 2.0 Hz, ArH), 6.43 (d, 2H, ^4J = 2.1 Hz, ArH) (**Figure S3a**). $^{13}C\{^1H\}$ NMR (100 MHz, $CDCl_3$, $CDCl_3$ = δ 77.0), δ, ppm: 24.5 ($\underline{CH_2}CH_2C(O)$), 25.5 (CH_2), 25.6 ($\underline{CH_2}C(O)N$), 28.4 (CH_2), 28.9 ($OCH_2\underline{CH_2}$), 30.8 ($\underline{CH_2}C(O)$), 54.9 (CH_2N_3), 67.8 (OCH_2), 101.0 (C_{arom}), 106.5 ($2C_{arom}$), 137.4 ($\underline{C_{arom}}$–CH_2N_3), 160.5 ($2\underline{C_{arom}}$–O), 168.6, 169.1 (C=O) (**Figure S3b**).

Synthesis of bis-hetero-α(2,6)sialic acid-galactose terminated azide 4 (Scheme S2). *N*,*N*-diisopropylethylamine (0.27 μL, 1.55×10^{-6} mol) was added to solution of 1S2S-11NC *N*-glycan (2.4 mg, 1.03×10^{-6} mol) and azide **3** (0.95 mg, 1.54×10^{-6} mol) in DMF (100 μL). After 4 hours of stirring at room temperature 1G2G–9NC *N*-glycan (1.8 mg, 1.03×10^{-6} mol) and *N*,*N*-diisopropylethylamine (0.27 μL, 1.55×10^{-6} mol) in DMF (50 μL) were added to the reaction mixture. The solution was stirred at room temperature for 20 hours under nitrogen atmosphere and then solvent was removed under vacuum. The residue was separated by HPLC [column: Hilic-phase Inertsil Hilic column (Amide, 4.6 x 150 mm); gradient: from

65% B / 35% A to 50% B / 50% A over 25 min; 1 mL/min; UV detection at 254 nm]. Bis-hetero azide **4** was eluted at 21.5 min. Yield: 0.92 mg (20%). ^{1}H NMR (400 MHz, D_2O, HOD = δ 4.79), δ, ppm: 1.30 – 1.54 (m, 8H, CH_2), 1.63 (quint, 4H, ^{3}J = 7.0 Hz, $\underline{CH_2}CH_2C(O)$), 1.67 – 1.83 (m, 6H, CH_2), 2.00 (s, 6H, CH_3), 2.03 (s, 6H, CH_3), 2.05 (s, 6H, CH_3), 2.07 (s, 12H, CH_3), 2.30 (t, 4H, ^{3}J = 7.0 Hz, $\underline{CH_2}C(O)$), 2.63 – 2.73 (m, 2H), 2.82 (d, 4H, *J* = 5.0 Hz), 3.41 – 4.05 (m, 124H), 4.06 – 4.12 (m, 6H), 4.20 (s, 1H), 4.26 (s, 1H), 4.38 (s, 1H), 4.46 (t, 3H, *J* = 8.3 Hz), 4.53 – 4.65 (m, 4H), 4.66 – 4.90 (m, including HOD signal), 4.94 (d, 2H, *J* = 7.9 Hz), 5.02 (d, 2H, *J* = 9.3 Hz), 5.14 (d, 2H, *J* = 5.0 Hz), 6.60 (bs, 1H, ArH), 6.66 (bs, 2H, ArH) (**Figure S4**). HRMS (ESI): detected: 1118.1619, calcd: 1118.1653 for $C_{175}H_{277}N_{17}O_{116}$ $[M–4H]^{4–}/4$.

Bis-homo-α(2,6)sialic acid terminated azide **15** was eluted at 24.5 min. Yield: 0.48 mg (9%). ^{1}H NMR (400 MHz, D_2O, HOD = δ 4.79), δ, ppm: 1.29 – 1.53 (m, 8H, CH_2), 1.62 (quint, 4H, ^{3}J = 7.1 Hz, $\underline{CH_2}CH_2C(O)$), 1.67 – 1.83 (m, 8H, CH_2), 2.00 (s, 6H, CH_3), 2.03 (s, 12H, CH_3), 2.07 (s, 18H, CH_3), 2.29 (t, 4H, ^{3}J = 7.1 Hz, $\underline{CH_2}C(O)$), 2.61 – 2.88 (m, 8H), 3.40 – 4.05 (m, 154H), 4.06 – 4.13 (m, 6H), 4.20 (s, 1H), 4.26 (s, 1H), 4.38 (s, 1H), 4.45 (d, 3H, *J* = 7.8 Hz), 4.51 – 4.65 (m, 7H), 4.67 – 5.09 (m, including HOD signal), 5.14 (s, 2H), 6.60 (bs, 1H, ArH), 6.67 (bs, 2H, ArH) (**Figure S5**). HRMS (ESI): detected: 1692.6029, calcd: 1692.6040 for $C_{197}H_{312}N_{19}NaO_{132}$ $[M–3H+Na]^{3–}/3$.

Bis-homo-galactose terminated azide **16** was eluted at 19.3 min. Yield: 0.57 mg (14%). ^{1}H NMR (400 MHz, D_2O, HOD = δ 4.79), δ, ppm: 1.29 – 1.48 (m, 8H, CH_2), 1.60 (quint, 4H, ^{3}J = 6.6 Hz, $\underline{CH_2}CH_2C(O)$), 1.75 (quint, 4H, ^{3}J = 5.8 Hz, $OCH_2\underline{CH_2}$), 1.97 (s, 6H, CH_3), 2.03 (s, 12H, CH_3), 2.05 (s, 6H, CH_3), 2.27 (t, 4H, ^{3}J = 6.6 Hz, $\underline{CH_2}C(O)$), 2.70 (s, 1H), 2.73 – 2.81 (m, 3H), 3.40 – 3.98 (m, 176H), 4.02 – 4.14 (m, 7H), 4.17 (s, 2H), 4.23 (s, 2H), 4.35 (s, 1H), 4.50 – 4.61 (m, 7H), 4.45 (d, 4H, *J* = 7.6 Hz), 4.56 (d, 4H, *J* = 6.3 Hz), 4.62 – 5.03 (m,

including HOD signal), 5.10 (s, 3H), 6.57 (bs, 1H, ArH), 6.64 (bs, 2H, ArH) (**Figure S6**). HRMS (ESI): detected: 1297.1560, calcd: 1297.1593 for $C_{153}H_{244}N_{15}O_{100}$ $[M–3H]^{3–}/3$.

Different mono-substituted products were not isolated as individual compounds.

Synthesis of bis-hetero-α(2,6)sialic acid-mannose terminated azide 5 (Scheme S3). Compound **5** was synthesized as described above for compound **4** from 1S2S-11NC *N*-glycan (4.1 mg, 1.7 × 10^{-6} mol), azide **3** (1.6 mg, 2.6 × 10^{-6} mol), *N,N*-diisopropylethylamine (0.9 μL, 5.2 × 10^{-6} mol) and 1M2M-5NC *N*-glycan (1.7 mg, 1.7 × 10^{-6} mol) in DMF (200 μL). Bis-hetero azide **5** was eluted with starting 1S2S-11NC *N*-glycan at the retention time 17.4 min. The fraction was reseparated by HPLC [column: Hilic-phase Inertsil Hilic column (Amide, 4.6 x 150 mm); gradient: 55% B / 45% A over 25 min; 1 mL/min; UV detection at 254 nm]. Bis-hetero azide **5** was eluted at 5.8 min. Yield: 1.7 mg (26%). ^{1}H NMR (400 MHz, D_2O, HOD = δ 4.79), δ, ppm: 1.23 – 1.46 (m, 8H, CH_2), 1.58 (quint, 4H, ^{3}J = 6.9 Hz, $\underline{CH_2}CH_2C(O)$), 1.64 – 1.77 (m, 6H, CH_2), 1.94 (s, 6H, CH_3), 1.98 (s, 6H, CH_3), 2.02 (s, 12H, CH_3), 2.25 (t, 4H, ^{3}J = 6.9 Hz, $\underline{CH_2}C(O)$), 2.56 – 3.00 (m, 3H), 3.40 – 4.09 (m, 126H), 4.10 – 4.12 (m, 4H), 4.12 – 4.45 (m, 2H), 4.50 –4.60 (m, 3H), 4.62 – 5.01 (m, including HOD signal), 5.07 (d, 2H, *J* = 13.8 Hz), 6.54 (bs, 1H, ArH), 6.61 (bs, 2H, ArH) (**Figure S7**). ESI-MS: detected: 1248.3, calcd: 1248.1 for $C_{147}H_{233}N_{15}O_{96}$: $[M–3H]^{3–}/3$.

Bis-homo-mannose terminated azide **17** was eluted at 19.3 min. Yield: 0.8 mg (19%). ^{1}H NMR (400 MHz, D_2O, HOD = δ 4.79): 1.28 – 1.49 (m, 8H, CH_2), 1.60 (quint, 4H, ^{3}J = 6.9 Hz, $\underline{CH_2}CH_2C(O)$), 1.75 (quint, 4H, ^{3}J = 6.2 Hz, $OCH_2\underline{CH_2}$), 1.97 (s, 6H, CH_3), 2.04 (s, 6H, CH_3), 2.27 (t, 4H, ^{3}J = 6.9 Hz, $\underline{CH_2}C(O)$), 2.70 (s, 5H), 2.72 – 2.86 (m, 2H), 3.40 – 3.98 (m, 96H), 4.02 – 4.11 (m, 8H), 4.24 (s, 1H), 4.35 (s, 1H), 4.51 – 5.05 (m, including HOD signal), 5.08 (bs, 4H), 6.57 (bs, 1H, ArH), 6.64 (bs, 2H, ArH) (**Figure S8**). ESI-MS: detected: 1216.50 calcd: 1216.47 for $C_{97}H_{153}N_{11}O_{60}$: $[M–2H]^{2–}/2$.

Different mono-substituted products were not isolated as individual compounds.

Synthesis of glycoalbumin 8a. Bis-hetero-α(2,6)sialic acid-galactose terminated azide **4** (0.72 mg, 0.16 μmol) in DMSO (38 μL) was added to 10 mM solution of cyclooctyne aldehyde[2] **1** in MeCN (14.6 μL, 0.15 μmol) under nitrogen atmosphere. The reaction mixture was heated to 70 °C and monitored by HPLC (column: Nacalai tesque column (5C18-AR-300, 4.6 x 250 mm); gradient: from 10% B / 90% A to 100% B over 30 min; 1 mL/min; UV detection at 254 nm). The clicked product **6** was detected at 16.6 min (ESI–HRMS: detected: 1673.9451, calcd: 1673.9447 for $C_{208}H_{306}N_{19}O_{122}$: $[M–3H]^{3–}/3$). After the consumption of starting aldehyde, the mixture was cooled down to r.t. and diluted with DMSO (155 μL) and water (465 μL). Subsequently, FL750–HSA[3] (155 μL, 9.7 nmol) was added and the resulting mixture was incubated overnight at 37 °C. The resulting solution was centrifuged through Amicon 10K® at 14.000 rpm for 10 min, and further washed with water three times to filter off any small molecules. The insoluble byproducts were further removed by filtering with Durapore PVDF 0.45 μm®. Resulting solution was diluted with water to give 194 μL solution of glycoalbumin **8a**. MALDI–TOF–MS (positive mode) detected the molecular weight of **8a** at 93.4 kDa, which contains average number, 4.6 molecules of bis-hetero-α(2,6)sialic acid-galactose terminated azide **4** per albumin (**Figure S9**).

Synthesis of glycoalbumin 8b. Solution of compound **6** prepared above (42.5 nmol, 5.0 eq) in DMSO (10 μL) was diluted with water (409 μL), DMSO (136 μL), and then solution of FL750–HSA[3] (136 μL, 8.5 nmol) was added. The mixture was incubated overnight at 37 °C. The purification procedure was the same as for **8a**. Resulting solution was diluted with water to give 170 μL solution of glycoalbumin **8b**. MALDI–TOF–MS (positive mode) detected the molecular weight of **8b** at 78.7 kDa, which contains average number, 1.7 molecules of bis-hetero-α(2,6)sialic acid-galactose terminated azide **4** per albumin (**Figure S10**).

Synthesis of glycoalbumin 9a. Bis-hetero-α(2,6)sialic acid-mannose terminated azide **5** (0.34 mg, 91 nmol) in DMSO (21 μL) was added 10 mM solution of cyclooctyne aldehyde[2] **1** in MeCN (8.2 μL, 82 nmol) under nitrogen atmosphere. The reaction mixture was heated to 70 °C and monitored by HPLC (column: Nacalai tesque column (5C18-AR-300, 4.6 x 250 mm); gradient: from 10% B / 90% A to 100% B over 30 min; 1 mL/min; UV detection at 254 nm). The clicked product **7** was detected at 17.1 min (ESI–MS: detected: 1073.1, calcd: 1072.9 for $C_{180}H_{259}N_{17}O_{102}$ $[M–4H]^{4–}/4$). After the consumption of the starting aldehyde, the mixture was cooled down to r.t. and diluted with DMSO (119 μL) and water (356 μL). Subsequently, FL750–HSA[3] (119 μL, 7.4 nmol) was added and the resulting mixture was incubated overnight at 37 °C. The purification procedure was the same as for **8a**. Resulting solution was diluted with water to give 148 μL solution of glycoalbumin **9a**. MALDI-TOF-MS (positive mode) detected the molecular weight of **9a** at 92.4 kDa, which contains average number, 5.2 molecules of bis-hetero-α(2,6)sialic acid-mannose terminated azide **5** per albumin (**Figure S11**).

Synthesis of glycoalbumin 9b. Solution of compound **7** prepared above (54.6 nmol, 7.0 eq) in DMSO (10 μL) was diluted with water (374 μL), DMSO (124 μL), and then solution of FL750–HSA[3] (124 μL, 7.8 nmol) was added. The mixture was incubated overnight at 37 °C. The purification procedure was the same as for **8a**. Resulting solution was diluted with water to give 156 μL solution of glycoalbumin **9b**. MALDI-TOF-MS (positive mode) detected the molecular weight of **9b** at 83.7 kDa, which contains average number, 2.4 molecules of bis-hetero-α(2,6)sialic acid-mannose terminated azide **5** per albumin (**Figure S12**).

References

[1] P. Antoni, D. Nyström, C. J. Hawker, A. Hult, M. Malkoch, *Chem. Commun.* **2007**, *22*,

2249.

[2] K. Tanaka, M. Kitadani, A. Tsutsui, A. R. Pradipta, R. Imamaki, S. Kitazume, N. Taniguchi, K. Fukase, *Org. Biomol. Chem.* **2014**, *12*, 1412.

[3] A. Ogura, T. Tahara, S. Nozaki, K. Morimoto, Y. Kizuka, S. Kitazume, M. Hara, S. Kojima, H. Onoe, A. Kurbangalieva, N. Taniguchi, Y. Watanabe, K. Tanaka, *Sci. Rep.* **2016**, *6*, 21797.

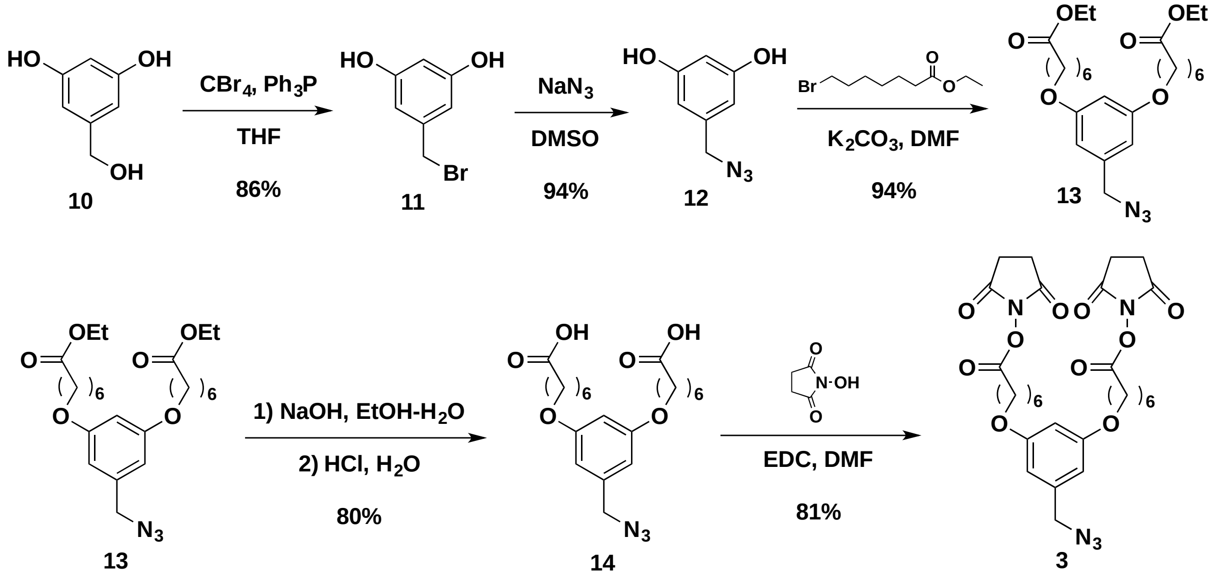

Scheme S1. Synthesis of bis-succinimidyl ester/azide **3**.

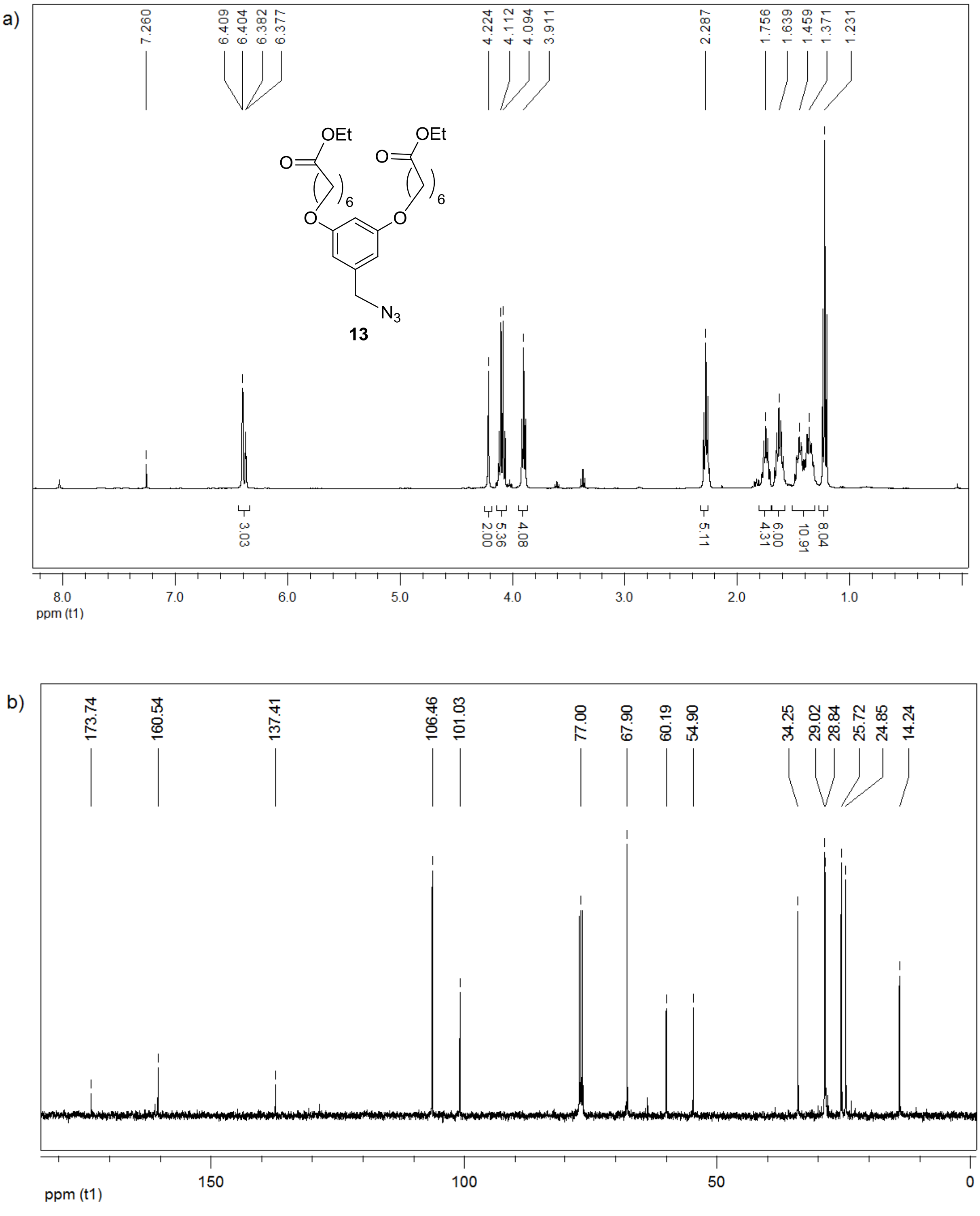

Figure S1. ^{1}H (a) and ^{13}C{^{1}H} (b) NMR spectra of compound **13** ($CDCl_3$).

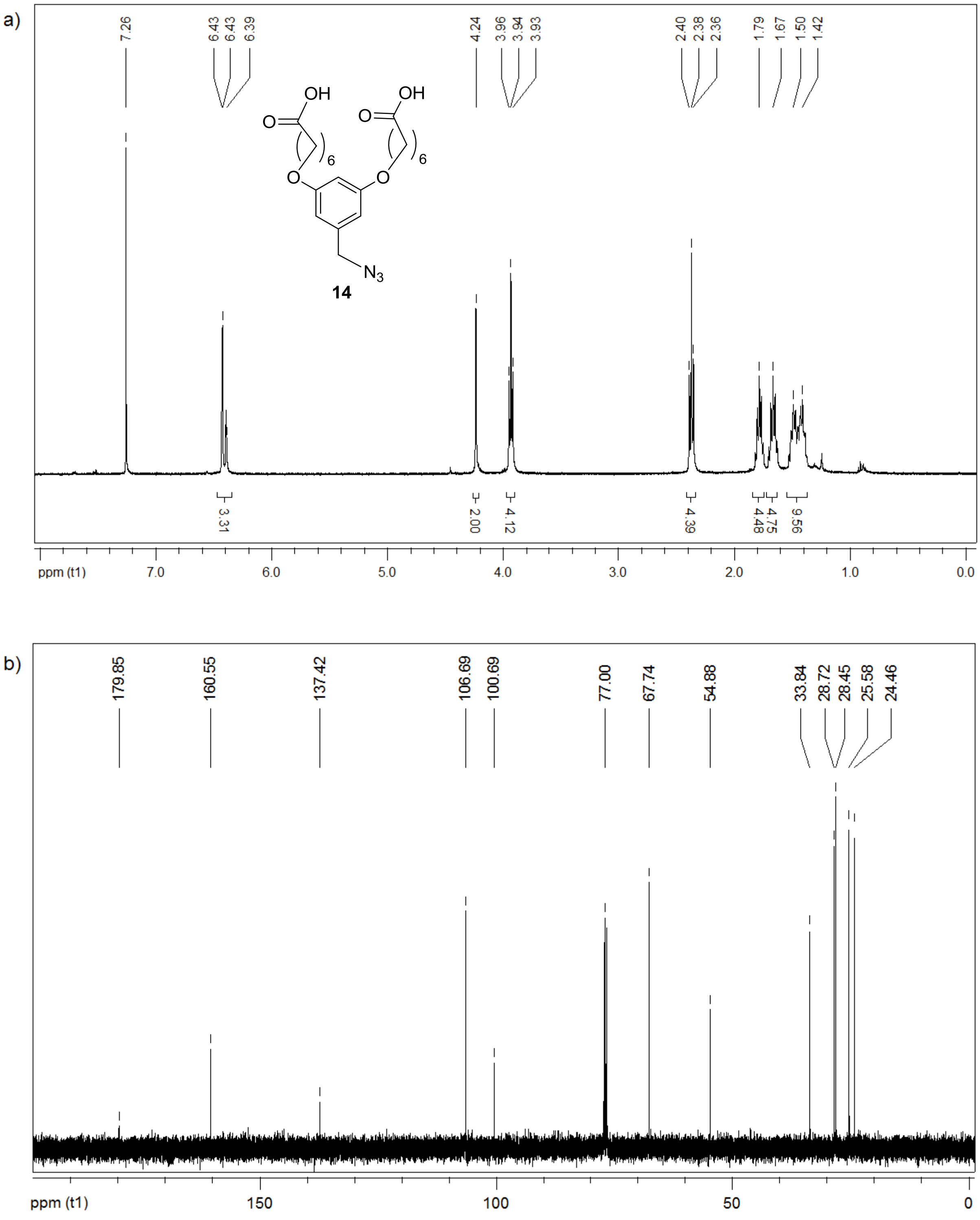

Figure S2. ^{1}H (a) and $^{13}C\{^{1}H\}$ (b) NMR spectra of compound **14** ($CDCl_3$).

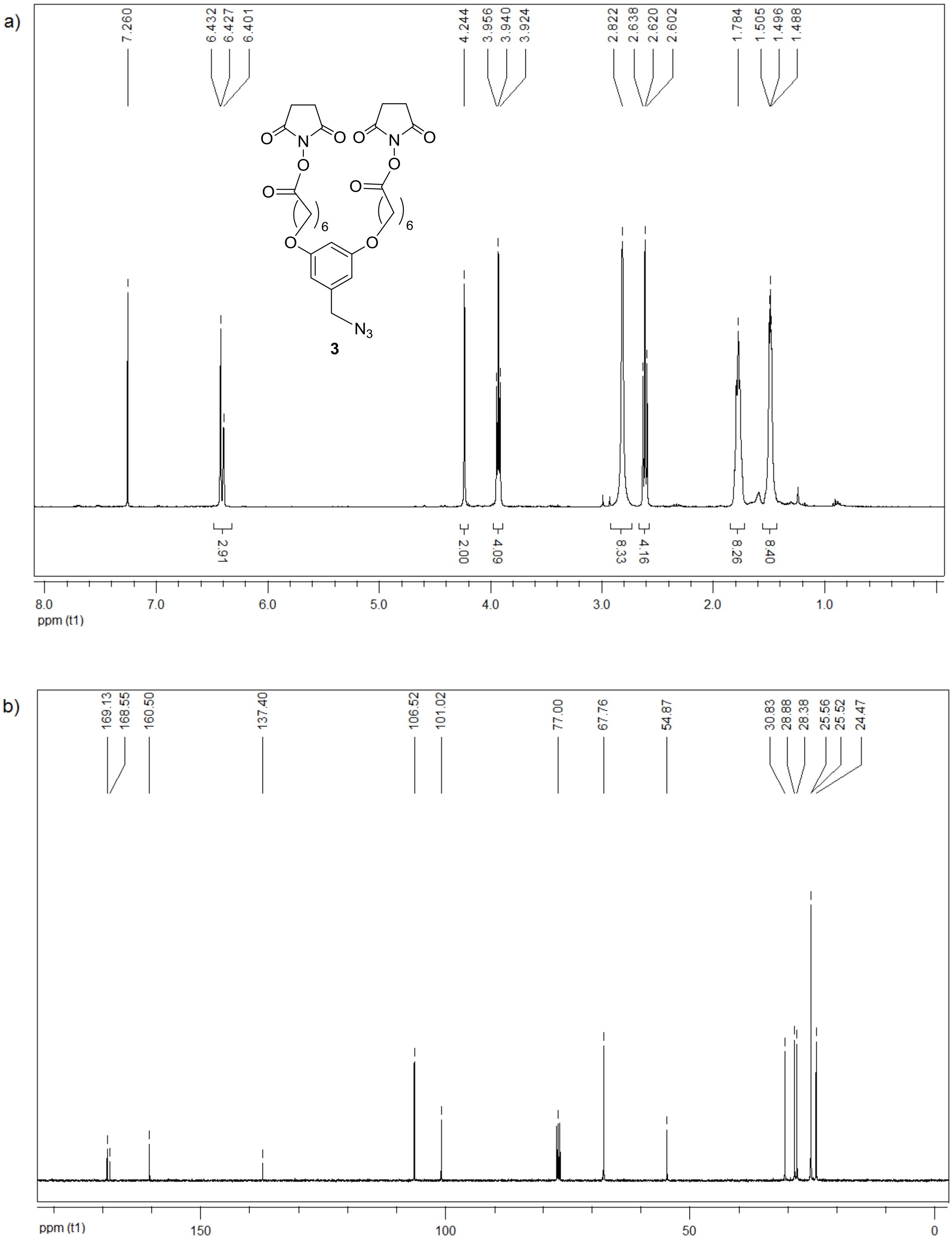

Figure S3. ^{1}H (a) and ^{13}C{^{1}H} (b) NMR spectra of compound **3** ($CDCl_3$).

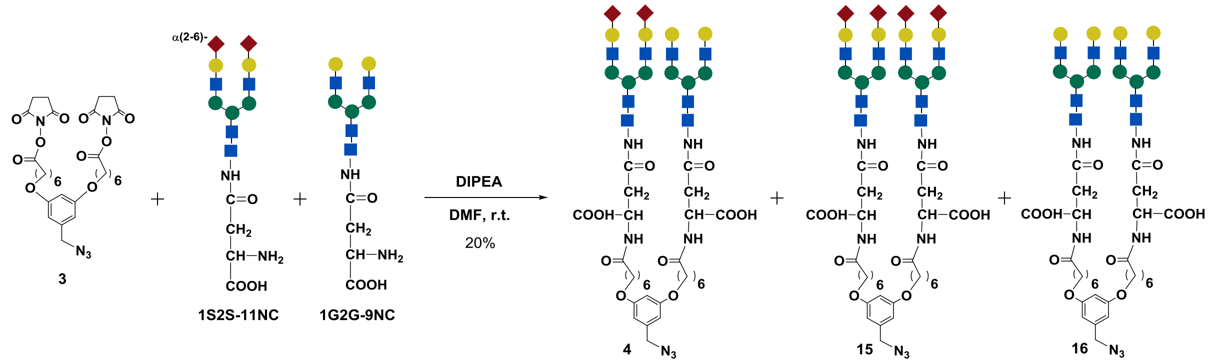

Scheme S2. Synthesis of bis-hetero-α(2,6)sialic acid-galactose terminated azide **4**.

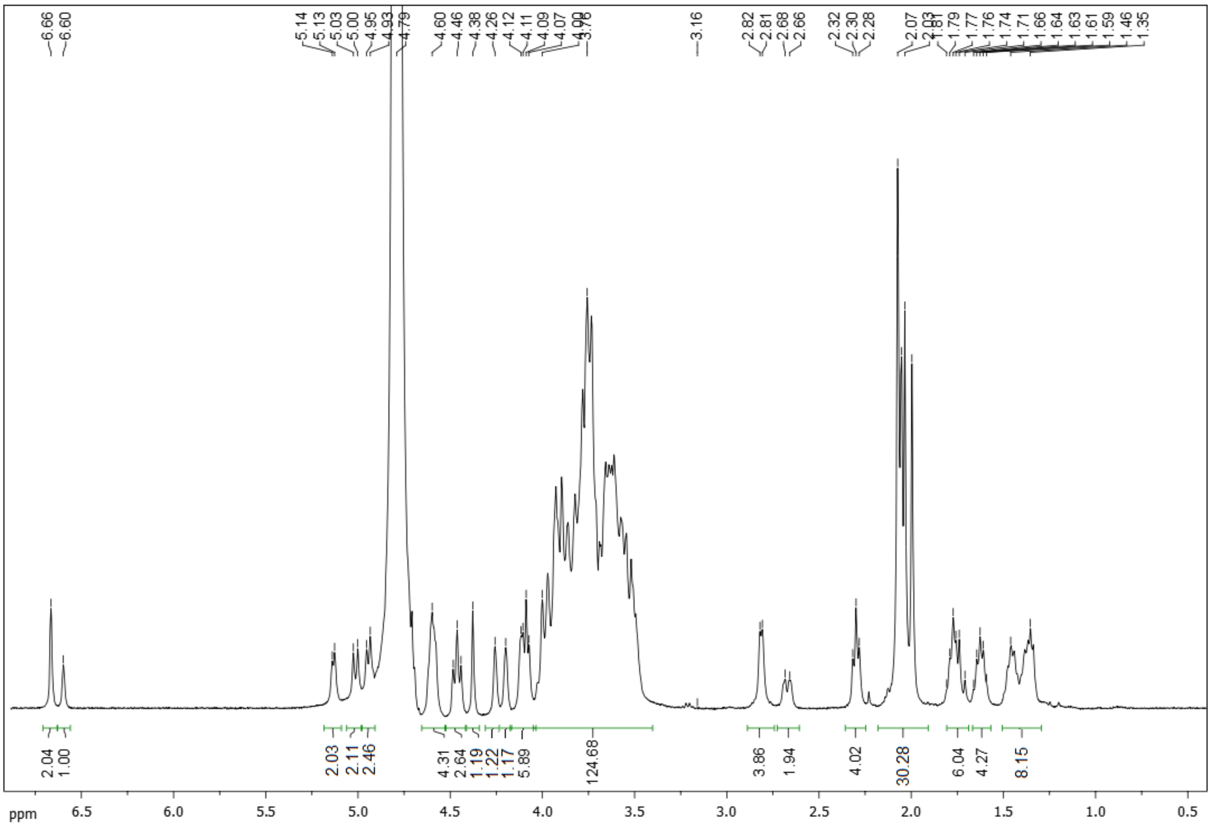

Figure S4. ^{1}H NMR spectrum of compound **4** (D_2O, 400 MHz).

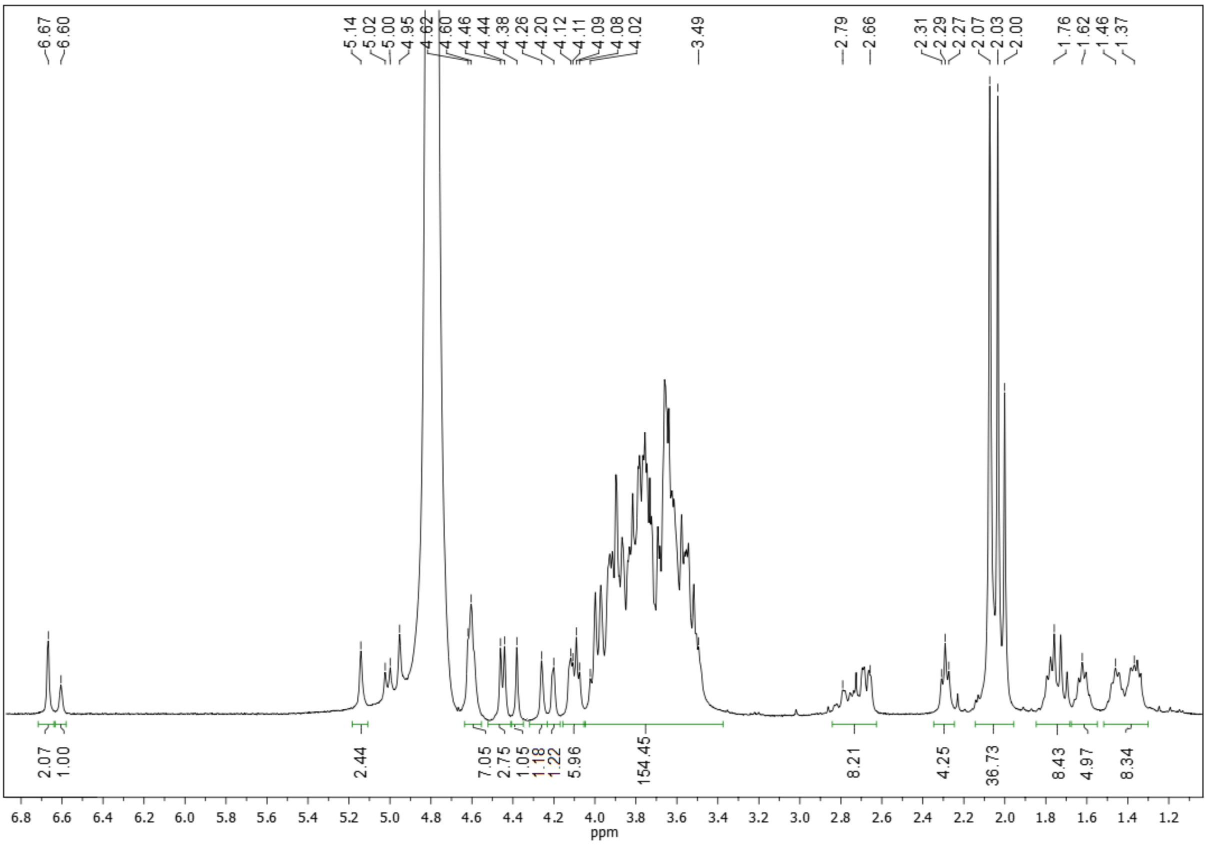

Figure S5. ^{1}H NMR spectrum of compound **15** (D_2O, 400 MHz).

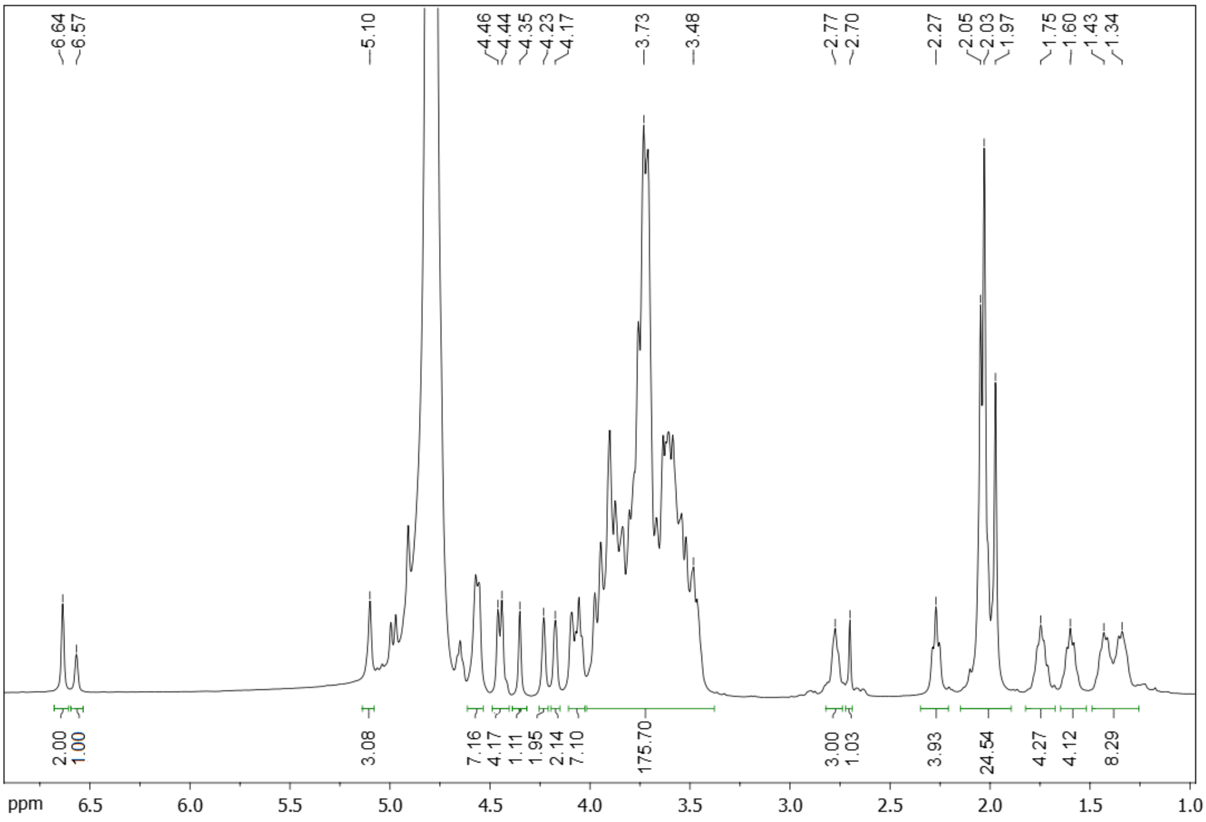

Figure S6. ^{1}H NMR spectrum of compound **16** (D_2O, 400 MHz).

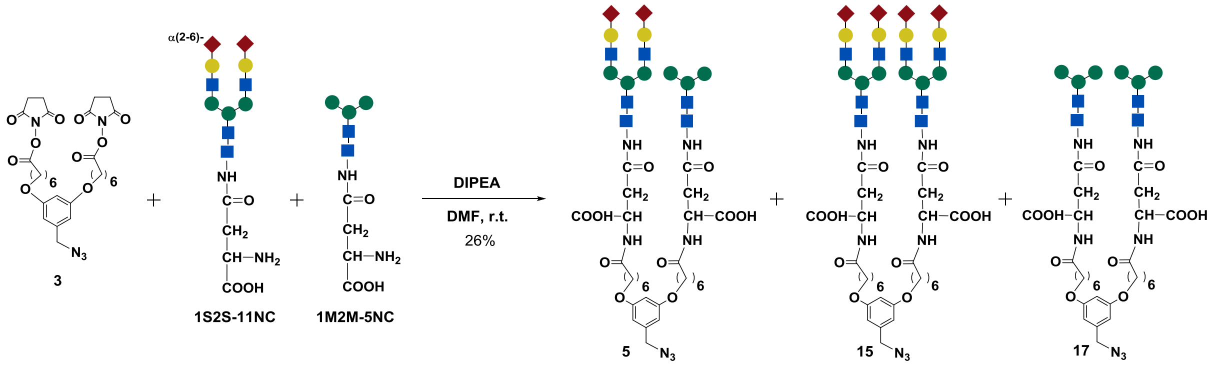

Scheme S3. Synthesis of bis-hetero-α(2,6)sialic acid-mannose terminated azide **5**.

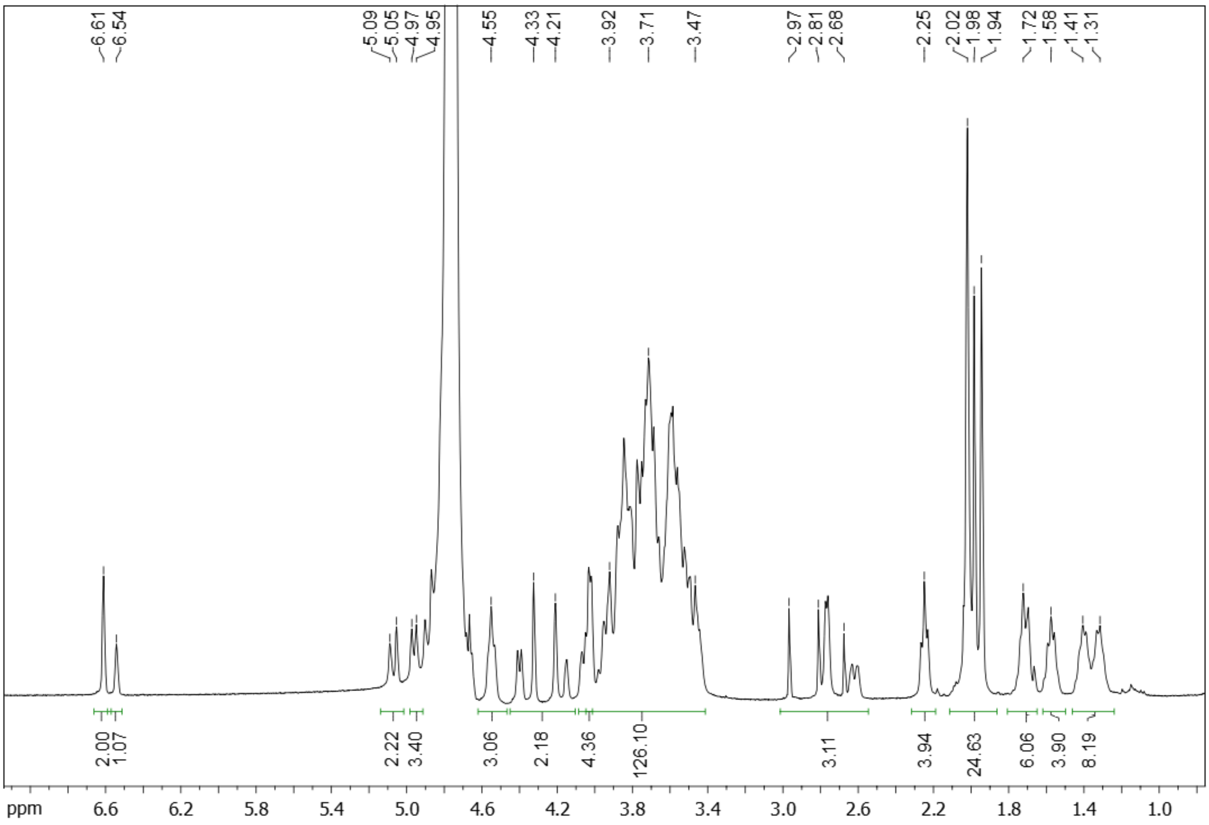

Figure S7. ^{1}H NMR spectrum of compound **5** (D_2O, 400 MHz).

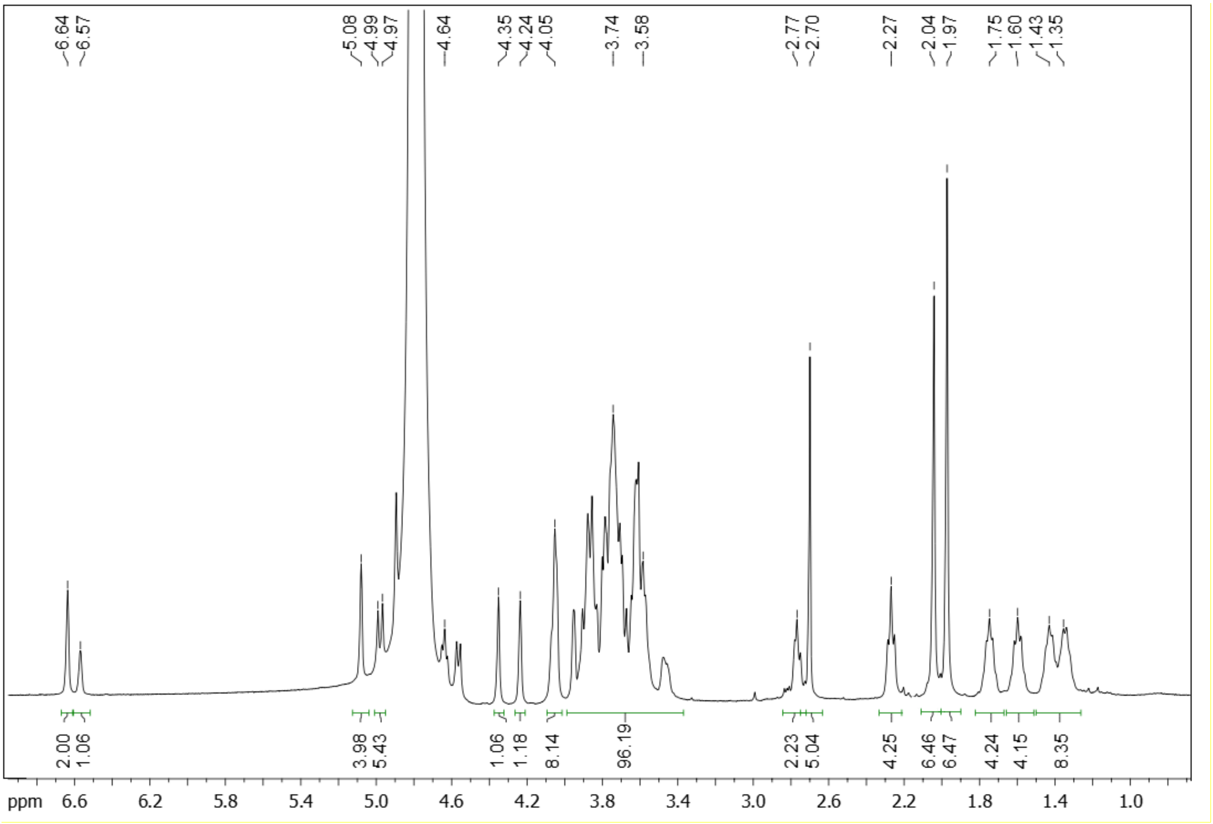

Figure S8. ^{1}H NMR spectrum of compound **17** (D_2O, 400 MHz).

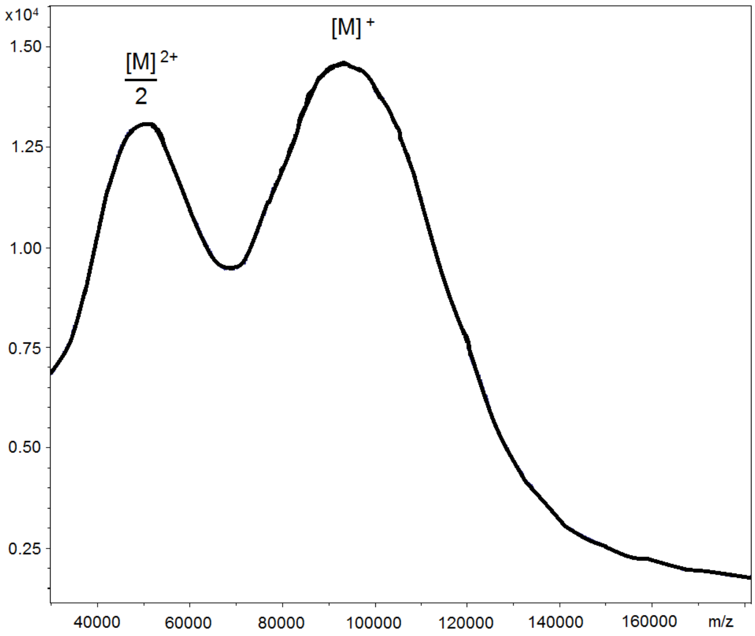

Figure S9. MALDI-TOF-MS of compound **8a** (positive mode).

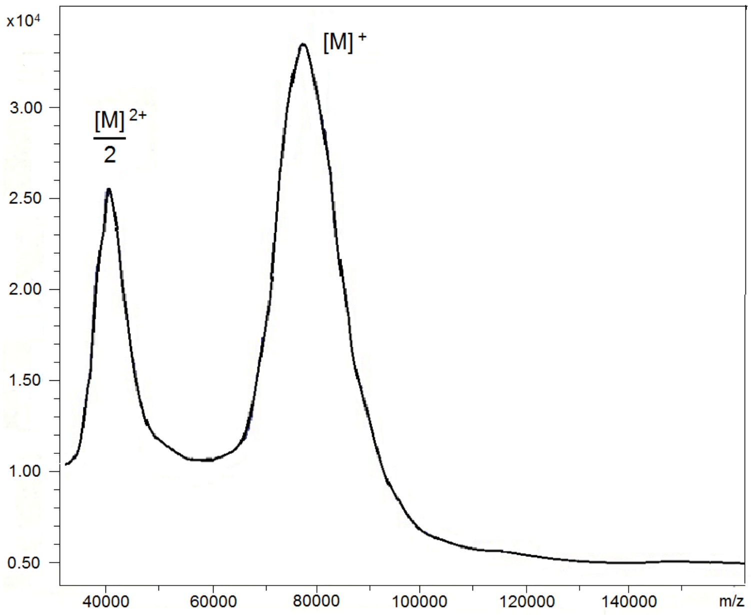

Figure S10. MALDI-TOF-MS of compound **8b** (positive mode).

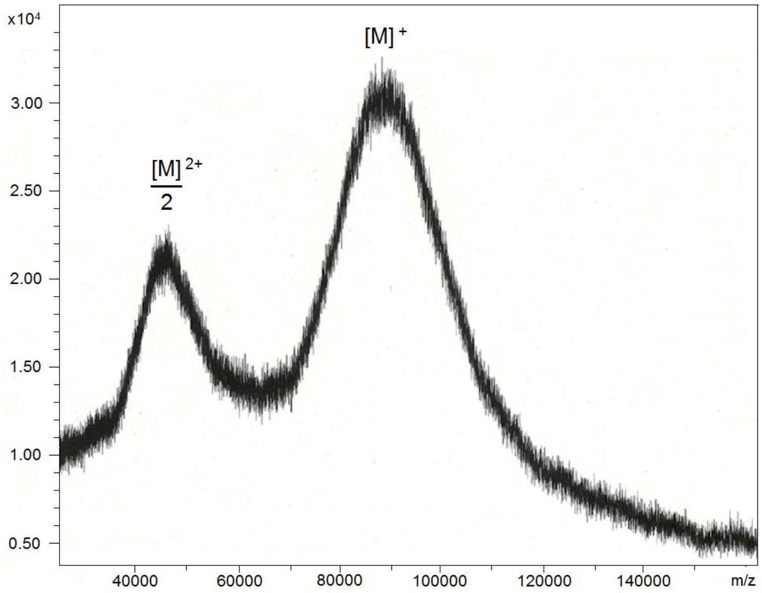

Figure S11. MALDI-TOF-MS of compound **9a** (positive mode).

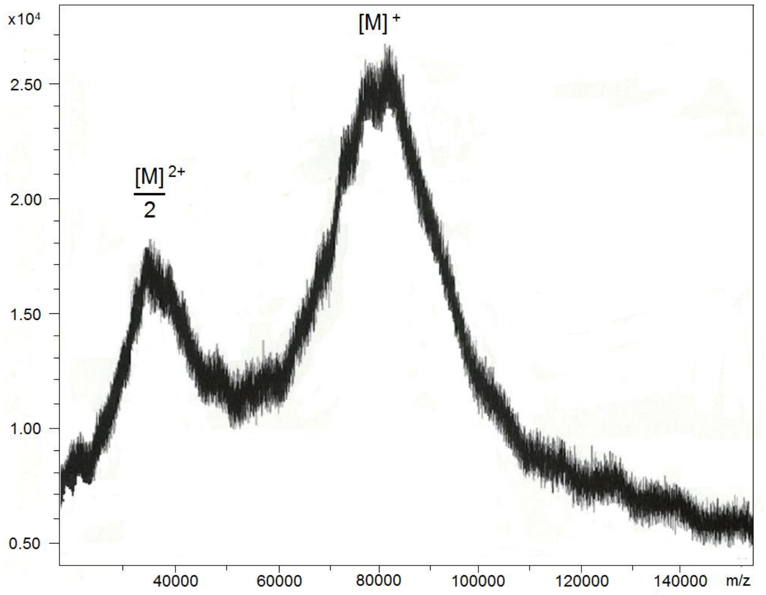

Figure S12. MALDI-TOF-MS of compound **9b** (positive mode).

Copyright WILEY-VCH Verlag GmbH & Co. KGaA, 69469 Weinheim, Germany, 2013.

Supporting Information

Sequential Double "Clicks" Toward Structurally Well-defined Heterogeneous *N*-Glycoclusters: The Importance of Cluster Heterogeneity on Pattern Recognition *In Vivo*

Liliya Latypova, Regina Sibgatullina, Akihiro Ogura, Katsumasa Fujiki, Alsu Khabibrakhmanova, Tsuyoshi Tahara, Satoshi Nozaki, Sayaka Urano, Kazuki Tsubokura, Hirotaka Onoe, Yasuyoshi Watanabe, Almira Kurbangalieva and Katsunori Tanaka**

Materials. 3,5-Dihydroxybenzyl alcohol, carbon tetrabromide, triphenylphosphine, sodium azide, *N*-hydroxysuccinimide (NHS) were purchased from Acros, ethyl 7-bromoheptanoate, human serum albumin (HSA) were obtained from Sigma-Aldrich, 1-ethyl-3-(3-(dimethylamino)propyl)carbodiimide hydrochloride (EDC) was provided by TCI, *N*-glycans were supplied from Glytech, Inc., fluorescent compound HiLyte^{TM} Fluor750 acid SE® was provided by AnaSpec, Inc. Fremont. Ultrapure water from Merck Milli-Q Advantage® was used for all synthetic experiments described in this paper.

High-resolution mass spectra (HRMS) were obtained on Bruker micrOTOF-QIII spectrometer® by electron spray ionization (ESI-TOF-MS). Mass spectra of the glycan-conjugated albumins were obtained on Bruker autoflex spectrometer® by matrix assisted laser desorption ionization (MALDI-TOF MS), using 2,5-dihydroxybenzoic acid as matrix. IR spectra of solid compounds were recorded on a Bruker Tensor-27 spectrometer from samples dispersed in Nujol and placed between KBr plates. ^{1}H and ^{13}C{^{1}H} NMR spectra were measured on Bruker Avance III 400 spectrometers (400.17 MHz for ^{1}H and 100.62 MHz for ^{13}C{^{1}H}) and JEOL RESONANCE AL400 (395.75 MHz for ^{1}H) in $CDCl_3$, acetone-d_6, D_2O at room temperature. The peak of the residual protonated solvent was used as the internal standard. Multiplicities are indicated as: s (singlet), bs (broad singlet), d (doublet), t (triplet), q

(quartet), quint (quintet), m (multiplet). Analytical thin layer chromatography (TLC) was carried out with silica gel plates «Merck» (silica gel 60, F254, supported on aluminium), using UV light as the visualizing agent. Column chromatography was performed on Silica gel 60 (Acros, 0.060–0.200 mm). Reverse and normal phase HPLC analysis/purification was performed on Shimadzu Prominence® system equipped with Nacalai tesque column (5C18-AR-300, 4.6 x 250 mm) and Hilic-phase Inertsil Hilic column (Amide, 4.6 x 150 mm) respectively. Two solvent systems, namely, A: H_2O containing 0.1% TFA and B: MeCN containing 0.1% TFA, were applied. The melting points were measured on an *OptiMelt Stanford Research Systems MPA100* automated melting point apparatus and were not corrected.

Synthesis of bis-succinimidyl ester/azide 3. The synthetic route includes 5 steps from the commercially available 3,5-dihydroxybenzyl alcohol **10** (**Scheme S1**). 5-(Bromomethyl)benzene-1,3-diol (**11**) and 5-(azidomethyl)benzene-1,3-diol (**12**) were synthesized according to the literature.[1]

Synthesis of diethyl 7,7'-((5-azidomethyl)-1,3-phenylene)bis(oxy)diheptanoate 13 (Scheme S1). Ethyl 7-bromoheptanoate (1.1 mL, 5.63 mmol) and K_2CO_3 (0.78 g, 5.63 mmol) were added to solution of azide **12** (0.31 g, 1.88 mmol) in DMF (15 mL). The reaction mixture was stirred for 17 hours at 80 °C under argon atmosphere. The mixture was cooled down to r.t. and then treated with 5% aqueous solution of citric acid (20 ml) at 0 °C. The resulting solution was extracted with ethyl acetate (2 x 20 mL). The combined organic layers were washed with water (3 x 10 mL), brine (30 mL) and dried under $MgSO_4$. The solvent was removed in vacuum to give product **13** as yellow oil. Yield: 0.84 g (94%), R_f 0.68 (EtOAc/dichloromethane, 1/7). IR (film), ν, cm^{-1}: 2979, 2939, 2863 (CH), 2100 (N_3), 1734 (C=O), 1597 (C=C). ^1H NMR (400 MHz, $CDCl_3$, $CHCl_3$ = δ 7.26), δ, ppm: 1.23 (t, 6H, ^3J = 7.1 Hz, CH_3), 1.29 – 1.51 (m, 8H, CH_2), 1.64 (quint, 4H, ^3J = 7.5 Hz, $\underline{CH_2}CH_2C(O)$), 1.76

(quint, 4H, ^3J = 6.5 Hz, $OCH_2\underline{CH_2}$), 2.29 (t, 4H, ^3J = 7.5 Hz, $\underline{CH_2}C(O)$), 3.91 (t, 4H, ^3J = 6.5 Hz, $O\underline{CH_2}$), 4.10 (q, 4H, ^3J = 7.1 Hz, $O\underline{CH_2}CH_3$), 4.22 (s, 2H, CH_2N_3), 6.38 (t, 1H, ^4J = 2.1 Hz, ArH), 6.40 (d, 2H, ^4J = 2.1 Hz, ArH) (**Figure S1a**). $^{13}C\{^1H\}$ NMR (100 MHz, $CDCl_3$, $CDCl_3$ = δ 77.0), δ, ppm: 14.2 (CH_3), 24.9 ($\underline{CH_2}CH_2C(O)$), 25.7, 28.8 (CH_2), 29.0 ($OCH_2\underline{CH_2}$), 34.3 ($\underline{CH_2}C(O)$), 54.9 (CH_2N_3), 60.2 ($O\underline{CH_2}CH_3$), 67.9 ($O\underline{CH_2}CH_2$), 101.0 (C_{arom}), 106.5 ($2C_{arom}$), 137.4 ($\underline{C_{arom}}$–CH_2N_3), 160.5 ($2\underline{C_{arom}}$–O), 173.7 (2C=O) (**Figure S1b**).

Synthesis of 7,7'-((5-azidomethyl)-1,3-phenylene)bis(oxy)diheptanoic acid 14 (Scheme S1). Solution of NaOH (0.23 g, 5.75 mmol) in water (5 mL) was added to solution of azide **13** (0.69 g, 1.44 mmol) in ethanol (35 mL). Reaction mixture was stirred at 40 °C for 9 hours and the solvents were removed under vacuum. The solid residue was dissolved in water (20 mL) and acidified with 5 M aq. HCl to pH 2. The solution was stirred at room temperature for 3 hours, extracted with ethyl acetate (2 x 25 mL) and then with trichloromethane (20 mL). The combined organic layers were dried under $MgSO_4$ and the solvent was evaporated to give yellow oil. Crystallization from tetrachloromethane yielded product **14** as colorless crystalline compound. Yield: 0.48 g (80%), R_f 0.44 (acetone/toluene, 1/2), m.p.: 71–72 °C. IR, ν, cm^{-1}: 3300 – 2600 broad (OH), 2118, 2094 (N_3), 1709 (C=O), 1606 (C=C). ^1H NMR (400 MHz, $CDCl_3$, $CHCl_3$ = δ 7.26), δ, ppm: 1.37 – 1.56 (m, 8H, CH_2), 1.68 (quint, 4H, ^3J = 7.3 Hz, $\underline{CH_2}CH_2C(O)$), 1.79 (quint, 4H, ^3J = 6.6 Hz, $OCH_2\underline{CH_2}$), 2.38 (t, 4H, ^3J = 7.3 Hz, $\underline{CH_2}C(O)$), 3.94 (t, 4H, ^3J = 6.6 Hz, $O\underline{CH_2}$), 4.24 (s, 2H, CH_2N_3), 6.40 (t, 1H, ^4J = 2.2 Hz, ArH), 6.43 (d, 2H, ^4J = 2.2 Hz, ArH) (**Figure S2a**). $^{13}C\{^1H\}$ NMR (100 MHz, $CDCl_3$, $CDCl_3$ = δ 77.0), δ, ppm: 24.5 ($\underline{CH_2}CH_2C(O)$), 25.6, 28.5 (CH_2), 28.7 ($OCH_2\underline{CH_2}$), 33.8 ($\underline{CH_2}C(O)$), 54.9 (CH_2N_3), 67.7 (OCH_2), 100.7 (C_{arom}), 106.7 ($2C_{arom}$), 137.43 ($\underline{C_{arom}}$–CH_2N_3), 160.6 ($2\underline{C_{arom}}$–O), 179.9 (2C=O) (**Figure S2b**).

Synthesis of di-*N*-hydroxysuccinimide 7,7'-((5-azidomethyl)-1,3-phenylene)bis(oxy)diheptanoate 3 (Scheme S1). *N*-hydroxysuccinimide (0.24 g, 2.09 mmol) and EDC hydrochloride (0.40 g, 2.09 mmol) were added to solution of compound **14** (0.38 g, 0.90 mmol) in DMF (15 mL) at 0 °C. Reaction mixture was stirred at room temperature for 22 hours under argon atmosphere. After the completion of the reaction, the mixture was cooled, washed with water (25 mL) and extracted with ethyl acetate (2 x 40 mL). The combined organic layers were dried under $MgSO_4$ and the solvent was evaporated. Resulting yellow oil was purified by column chromatography (eluent ethanol/dichloromethane, 1/20) to give product **3** as a colorless solid. Yield: 0.45 g (81%), R_f 0.86 (ethanol/dichloromethane, 1/20), m.p.: 54 °C. IR, ν, cm^{-1}: 2102 (N_3), 1813, 1784, 1741 (C=O), 1596 (C=C). ^1H NMR (400 MHz, $CDCl_3$, $CHCl_3$ = δ 7.26), δ, ppm: 1.41 – 1.57 (m, 8H, CH_2), 1.69 – 1.86 (m, 8H, CH_2), 2.62 (t, 4H, ^3J = 7.4 Hz, $\underline{CH_2}$C(O)), 2.82 (bs, 8H, CH_2C(O)N), 3.94 (t, 4H, ^3J = 6.4 Hz, O$\underline{CH_2}$), 4.24 (s, 2H, CH_2N_3), 6.40 (t, 1H, ^4J = 2.0 Hz, ArH), 6.43 (d, 2H, ^4J = 2.1 Hz, ArH) (**Figure S3a**). $^{13}C\{^1H\}$ NMR (100 MHz, $CDCl_3$, $CDCl_3$ = δ 77.0), δ, ppm: 24.5 ($\underline{CH_2}CH_2$C(O)), 25.5 (CH_2), 25.6 ($\underline{CH_2}$C(O)N), 28.4 (CH_2), 28.9 ($OCH_2\underline{CH_2}$), 30.8 ($\underline{CH_2}$C(O)), 54.9 (CH_2N_3), 67.8 (OCH_2), 101.0 (C_{arom}), 106.5 (2C_{arom}), 137.4 ($\underline{C_{arom}}$–CH_2N_3), 160.5 (2$\underline{C_{arom}}$–O), 168.6, 169.1 (C=O) (**Figure S3b**).

Synthesis of bis-hetero-α(2,6)sialic acid-galactose terminated azide 4 (Scheme S2). *N*,*N*-diisopropylethylamine (0.27 µL, 1.55 × 10^{-6} mol) was added to solution of 1S2S-11NC *N*-glycan (2.4 mg, 1.03 × 10^{-6} mol) and azide **3** (0.95 mg, 1.54 × 10^{-6} mol) in DMF (100 µL). After 4 hours of stirring at room temperature 1G2G–9NC *N*-glycan (1.8 mg, 1.03 × 10^{-6} mol) and *N*,*N*-diisopropylethylamine (0.27 µL, 1.55 × 10^{-6} mol) in DMF (50 µL) were added to the reaction mixture. The solution was stirred at room temperature for 20 hours under nitrogen atmosphere and then solvent was removed under vacuum. The residue was separated by HPLC [column: Hilic-phase Inertsil Hilic column (Amide, 4.6 x 150 mm); gradient: from

65% B / 35% A to 50% B / 50% A over 25 min; 1 mL/min; UV detection at 254 nm]. Bis-hetero azide **4** was eluted at 21.5 min. Yield: 0.92 mg (20%). ^{1}H NMR (400 MHz, D_2O, HOD = δ 4.79), δ, ppm: 1.30 – 1.54 (m, 8H, CH_2), 1.63 (quint, 4H, ^3J = 7.0 Hz, $\underline{CH_2}CH_2C(O)$), 1.67 – 1.83 (m, 6H, CH_2), 2.00 (s, 6H, CH_3), 2.03 (s, 6H, CH_3), 2.05 (s, 6H, CH_3), 2.07 (s, 12H, CH_3), 2.30 (t, 4H, ^3J = 7.0 Hz, $\underline{CH_2}C(O)$), 2.63 – 2.73 (m, 2H), 2.82 (d, 4H, J = 5.0 Hz), 3.41 – 4.05 (m, 124H), 4.06 – 4.12 (m, 6H), 4.20 (s, 1H), 4.26 (s, 1H), 4.38 (s, 1H), 4.46 (t, 3H, J = 8.3 Hz), 4.53 – 4.65 (m, 4H), 4.66 – 4.90 (m, including HOD signal), 4.94 (d, 2H, J = 7.9 Hz), 5.02 (d, 2H, J = 9.3 Hz), 5.14 (d, 2H, J = 5.0 Hz), 6.60 (bs, 1H, ArH), 6.66 (bs, 2H, ArH) (**Figure S4**). HRMS (ESI): detected: 1118.1619, calcd: 1118.1653 for $C_{175}H_{277}N_{17}O_{116}$ $[M–4H]^{4–}/4$.

Bis-homo-α(2,6)sialic acid terminated azide **15** was eluted at 24.5 min. Yield: 0.48 mg (9%). ^{1}H NMR (400 MHz, D_2O, HOD = δ 4.79), δ, ppm: 1.29 – 1.53 (m, 8H, CH_2), 1.62 (quint, 4H, ^3J = 7.1 Hz, $\underline{CH_2}CH_2C(O)$), 1.67 – 1.83 (m, 8H, CH_2), 2.00 (s, 6H, CH_3), 2.03 (s, 12H, CH_3), 2.07 (s, 18H, CH_3), 2.29 (t, 4H, ^3J = 7.1 Hz, $\underline{CH_2}C(O)$), 2.61 – 2.88 (m, 8H), 3.40 – 4.05 (m, 154H), 4.06 – 4.13 (m, 6H), 4.20 (s, 1H), 4.26 (s, 1H), 4.38 (s, 1H), 4.45 (d, 3H, J = 7.8 Hz), 4.51 – 4.65 (m, 7H), 4.67 – 5.09 (m, including HOD signal), 5.14 (s, 2H), 6.60 (bs, 1H, ArH), 6.67 (bs, 2H, ArH) (**Figure S5**). HRMS (ESI): detected: 1692.6029, calcd: 1692.6040 for $C_{197}H_{312}N_{19}NaO_{132}$ $[M–3H+Na]^{3–}/3$.

Bis-homo-galactose terminated azide **16** was eluted at 19.3 min. Yield: 0.57 mg (14%). ^{1}H NMR (400 MHz, D_2O, HOD = δ 4.79), δ, ppm: 1.29 – 1.48 (m, 8H, CH_2), 1.60 (quint, 4H, ^3J = 6.6 Hz, $\underline{CH_2}CH_2C(O)$), 1.75 (quint, 4H, ^3J = 5.8 Hz, $OCH_2\underline{CH_2}$), 1.97 (s, 6H, CH_3), 2.03 (s, 12H, CH_3), 2.05 (s, 6H, CH_3), 2.27 (t, 4H, ^3J = 6.6 Hz, $\underline{CH_2}C(O)$), 2.70 (s, 1H), 2.73 – 2.81 (m, 3H), 3.40 – 3.98 (m, 176H), 4.02 – 4.14 (m, 7H), 4.17 (s, 2H), 4.23 (s, 2H), 4.35 (s, 1H), 4.50 – 4.61 (m, 7H), 4.45 (d, 4H, J = 7.6 Hz), 4.56 (d, 4H, J = 6.3 Hz), 4.62 – 5.03 (m,

including HOD signal), 5.10 (s, 3H), 6.57 (bs, 1H, ArH), 6.64 (bs, 2H, ArH) (**Figure S6**). HRMS (ESI): detected: 1297.1560, calcd: 1297.1593 for $C_{153}H_{244}N_{15}O_{100}$ $[M–3H]^{3–}/3$.

Different mono-substituted products were not isolated as individual compounds.

Synthesis of bis-hetero-α(2,6)sialic acid-mannose terminated azide 5 (Scheme S3). Compound **5** was synthesized as described above for compound **4** from 1S2S-11NC *N*-glycan (4.1 mg, 1.7×10^{-6} mol), azide **3** (1.6 mg, 2.6×10^{-6} mol), *N,N*-diisopropylethylamine (0.9 μL, 5.2×10^{-6} mol) and 1M2M-5NC *N*-glycan (1.7 mg, 1.7×10^{-6} mol) in DMF (200 μL). Bis-hetero azide **5** was eluted with starting 1S2S-11NC *N*-glycan at the retention time 17.4 min. The fraction was reseparated by HPLC [column: Hilic-phase Inertsil Hilic column (Amide, 4.6 x 150 mm); gradient: 55% B / 45% A over 25 min; 1 mL/min; UV detection at 254 nm]. Bis-hetero azide **5** was eluted at 5.8 min. Yield: 1.7 mg (26%). ^{1}H NMR (400 MHz, D_2O, HOD = δ 4.79), δ, ppm: 1.23 – 1.46 (m, 8H, CH_2), 1.58 (quint, 4H, ^{3}J = 6.9 Hz, $\underline{CH_2}CH_2C(O)$), 1.64 – 1.77 (m, 6H, CH_2), 1.94 (s, 6H, CH_3), 1.98 (s, 6H, CH_3), 2.02 (s, 12H, CH_3), 2.25 (t, 4H, ^{3}J = 6.9 Hz, $\underline{CH_2}C(O)$), 2.56 – 3.00 (m, 3H), 3.40 – 4.09 (m, 126H), 4.10 – 4.12 (m, 4H), 4.12 – 4.45 (m, 2H), 4.50 –4.60 (m, 3H), 4.62 – 5.01 (m, including HOD signal), 5.07 (d, 2H, *J* = 13.8 Hz), 6.54 (bs, 1H, ArH), 6.61 (bs, 2H, ArH) (**Figure S7**). ESI-MS: detected: 1248.3, calcd: 1248.1 for $C_{147}H_{233}N_{15}O_{96}$: $[M–3H]^{3–}/3$.

Bis-homo-mannose terminated azide **17** was eluted at 19.3 min. Yield: 0.8 mg (19%). ^{1}H NMR (400 MHz, D_2O, HOD = δ 4.79): 1.28 – 1.49 (m, 8H, CH_2), 1.60 (quint, 4H, ^{3}J = 6.9 Hz, $\underline{CH_2}CH_2C(O)$), 1.75 (quint, 4H, ^{3}J = 6.2 Hz, $OCH_2\underline{CH_2}$), 1.97 (s, 6H, CH_3), 2.04 (s, 6H, CH_3), 2.27 (t, 4H, ^{3}J = 6.9 Hz, $\underline{CH_2}C(O)$), 2.70 (s, 5H), 2.72 – 2.86 (m, 2H), 3.40 – 3.98 (m, 96H), 4.02 – 4.11 (m, 8H), 4.24 (s, 1H), 4.35 (s, 1H), 4.51 – 5.05 (m, including HOD signal), 5.08 (bs, 4H), 6.57 (bs, 1H, ArH), 6.64 (bs, 2H, ArH) (**Figure S8**). ESI-MS: detected: 1216.50 calcd: 1216.47 for $C_{97}H_{153}N_{11}O_{60}$: $[M–2H]^{2–}/2$.

Different mono-substituted products were not isolated as individual compounds.

Synthesis of glycoalbumin 8a. Bis-hetero-α(2,6)sialic acid-galactose terminated azide **4** (0.72 mg, 0.16 μmol) in DMSO (38 μL) was added to 10 mM solution of cyclooctyne aldehyde[2] **1** in MeCN (14.6 μL, 0.15 μmol) under nitrogen atmosphere. The reaction mixture was heated to 70 °C and monitored by HPLC (column: Nacalai tesque column (5C18-AR-300, 4.6 x 250 mm); gradient: from 10% B / 90% A to 100% B over 30 min; 1 mL/min; UV detection at 254 nm). The clicked product **6** was detected at 16.6 min (ESI–HRMS: detected: 1673.9451, calcd: 1673.9447 for $C_{208}H_{306}N_{19}O_{122}$: $[M–3H]^{3–}/3$). After the consumption of starting aldehyde, the mixture was cooled down to r.t. and diluted with DMSO (155 μL) and water (465 μL). Subsequently, FL750–HSA[3] (155 μL, 9.7 nmol) was added and the resulting mixture was incubated overnight at 37 °C. The resulting solution was centrifuged through Amicon 10K® at 14.000 rpm for 10 min, and further washed with water three times to filter off any small molecules. The insoluble byproducts were further removed by filtering with Durapore PVDF 0.45 μm®. Resulting solution was diluted with water to give 194 μL solution of glycoalbumin **8a**. MALDI–TOF–MS (positive mode) detected the molecular weight of **8a** at 93.4 kDa, which contains average number, 4.6 molecules of bis-hetero-α(2,6)sialic acid-galactose terminated azide **4** per albumin (**Figure S9**).

Synthesis of glycoalbumin 8b. Solution of compound **6** prepared above (42.5 nmol, 5.0 eq) in DMSO (10 μL) was diluted with water (409 μL), DMSO (136 μL), and then solution of FL750–HSA[3] (136 μL, 8.5 nmol) was added. The mixture was incubated overnight at 37 °C. The purification procedure was the same as for **8a**. Resulting solution was diluted with water to give 170 μL solution of glycoalbumin **8b**. MALDI–TOF–MS (positive mode) detected the molecular weight of **8b** at 78.7 kDa, which contains average number, 1.7 molecules of bis-hetero-α(2,6)sialic acid-galactose terminated azide **4** per albumin (**Figure S10**).

Synthesis of glycoalbumin 9a. Bis-hetero-α(2,6)sialic acid-mannose terminated azide **5** (0.34 mg, 91 nmol) in DMSO (21 μL) was added 10 mM solution of cyclooctyne aldehyde[2] **1** in MeCN (8.2 μL, 82 nmol) under nitrogen atmosphere. The reaction mixture was heated to 70 °C and monitored by HPLC (column: Nacalai tesque column (5C18-AR-300, 4.6 x 250 mm); gradient: from 10% B / 90% A to 100% B over 30 min; 1 mL/min; UV detection at 254 nm). The clicked product **7** was detected at 17.1 min (ESI–MS: detected: 1073.1, calcd: 1072.9 for $C_{180}H_{259}N_{17}O_{102}$ $[M–4H]^{4–}/4$). After the consumption of the starting aldehyde, the mixture was cooled down to r.t. and diluted with DMSO (119 μL) and water (356 μL). Subsequently, FL750–HSA[3] (119 μL, 7.4 nmol) was added and the resulting mixture was incubated overnight at 37 °C. The purification procedure was the same as for **8a**. Resulting solution was diluted with water to give 148 μL solution of glycoalbumin **9a**. MALDI-TOF-MS (positive mode) detected the molecular weight of **9a** at 92.4 kDa, which contains average number, 5.2 molecules of bis-hetero-α(2,6)sialic acid-mannose terminated azide **5** per albumin (**Figure S11**).

Synthesis of glycoalbumin 9b. Solution of compound **7** prepared above (54.6 nmol, 7.0 eq) in DMSO (10 μL) was diluted with water (374 μL), DMSO (124 μL), and then solution of FL750–HSA[3] (124 μL, 7.8 nmol) was added. The mixture was incubated overnight at 37 °C. The purification procedure was the same as for **8a**. Resulting solution was diluted with water to give 156 μL solution of glycoalbumin **9b**. MALDI-TOF-MS (positive mode) detected the molecular weight of **9b** at 83.7 kDa, which contains average number, 2.4 molecules of bis-hetero-α(2,6)sialic acid-mannose terminated azide **5** per albumin (**Figure S12**).

References

[1] P. Antoni, D. Nyström, C. J. Hawker, A. Hult, M. Malkoch, *Chem. Commun.* **2007**, *22*,

2249.

[2] K. Tanaka, M. Kitadani, A. Tsutsui, A. R. Pradipta, R. Imamaki, S. Kitazume, N. Taniguchi, K. Fukase, *Org. Biomol. Chem.* **2014**, *12*, 1412.

[3] A. Ogura, T. Tahara, S. Nozaki, K. Morimoto, Y. Kizuka, S. Kitazume, M. Hara, S. Kojima, H. Onoe, A. Kurbangalieva, N. Taniguchi, Y. Watanabe, K. Tanaka, *Sci. Rep.* **2016**, *6*, 21797.

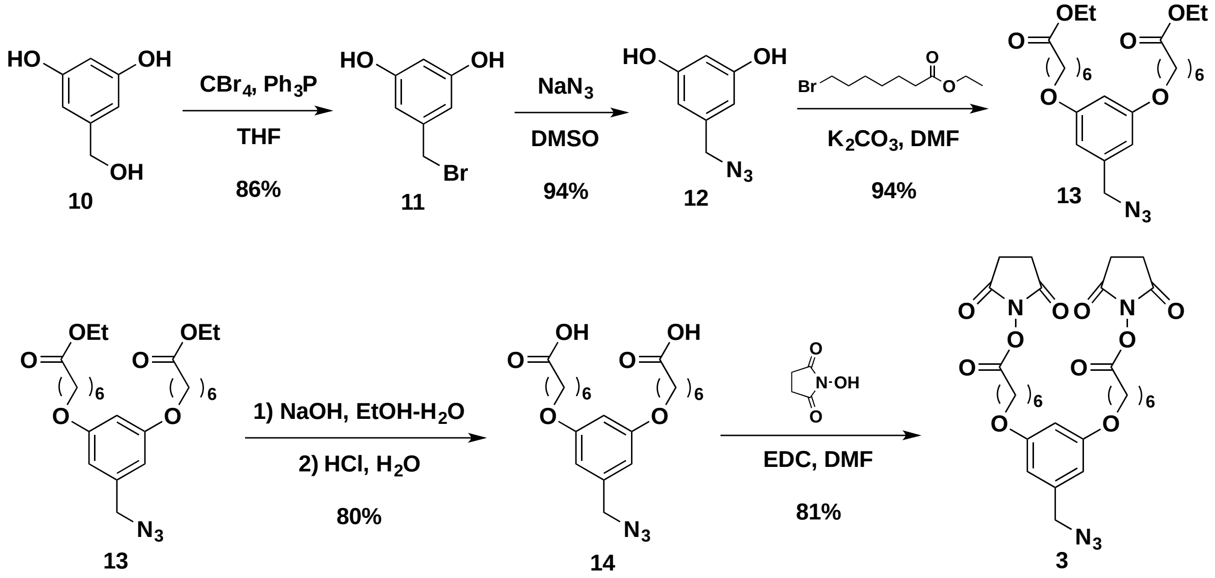

Scheme S1. Synthesis of bis-succinimidyl ester/azide **3**.

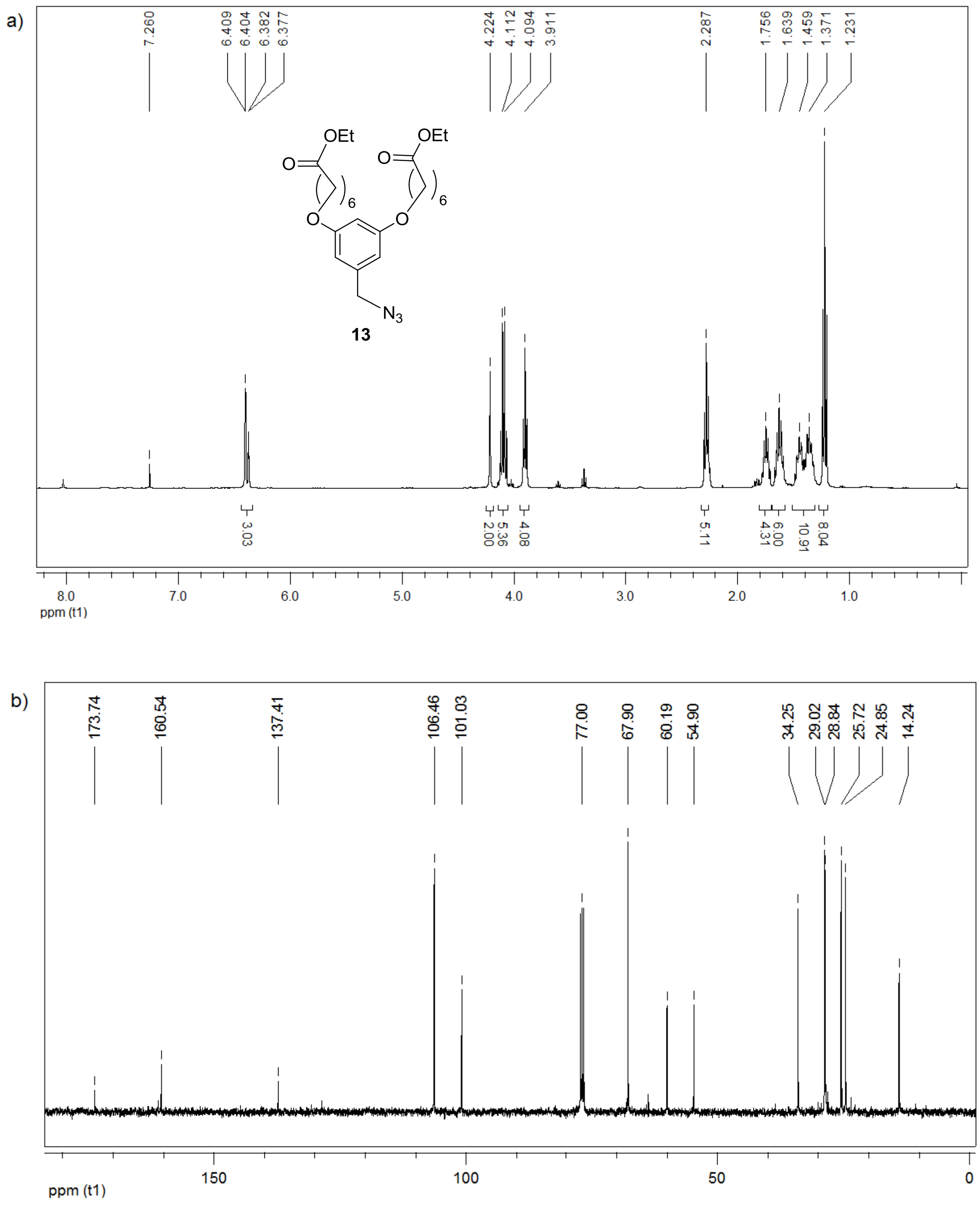

Figure S1. ^{1}H (a) and $^{13}C\{^{1}H\}$ (b) NMR spectra of compound **13** ($CDCl_3$).

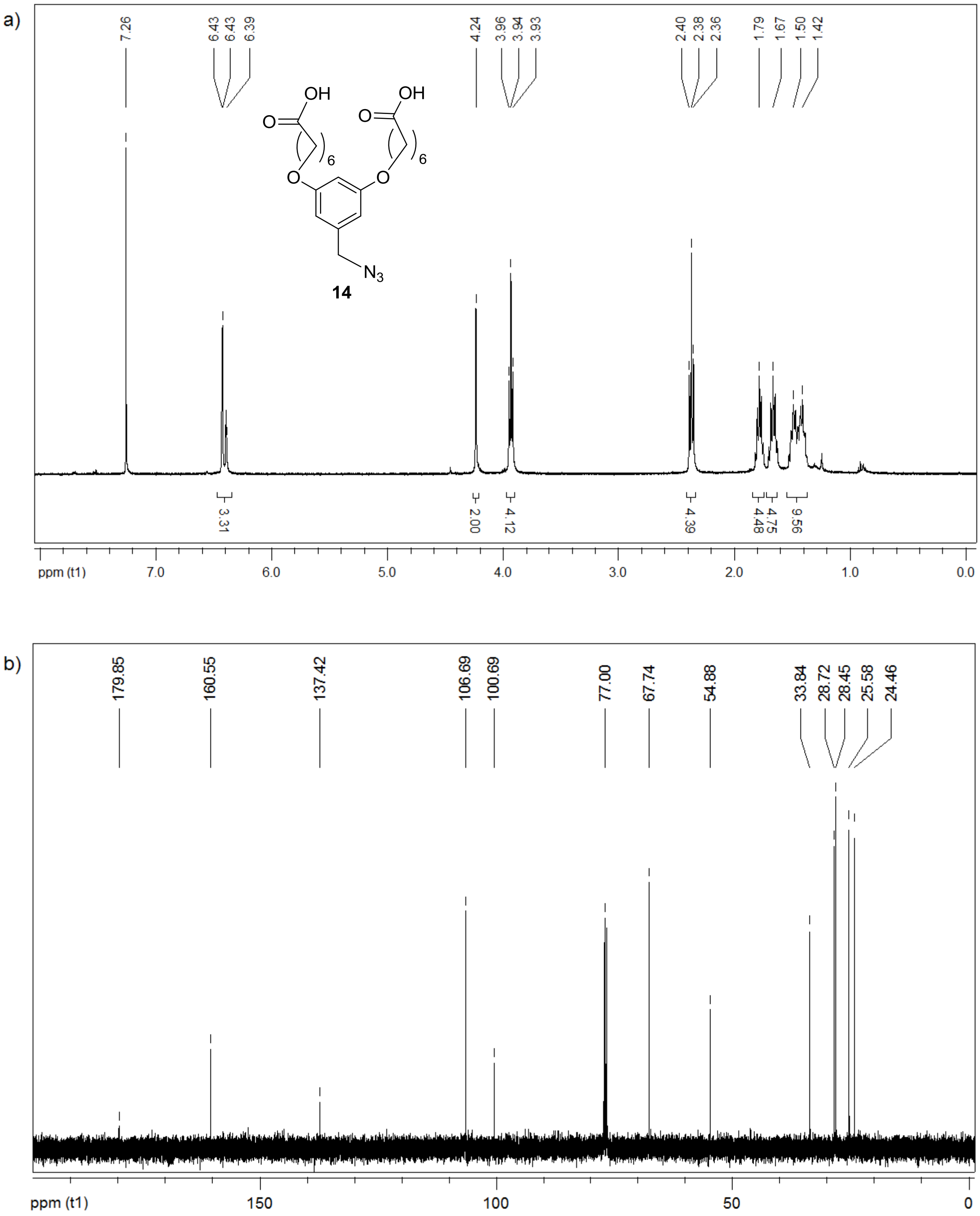

Figure S2. ^{1}H (a) and $^{13}C\{^{1}H\}$ (b) NMR spectra of compound **14** ($CDCl_3$).

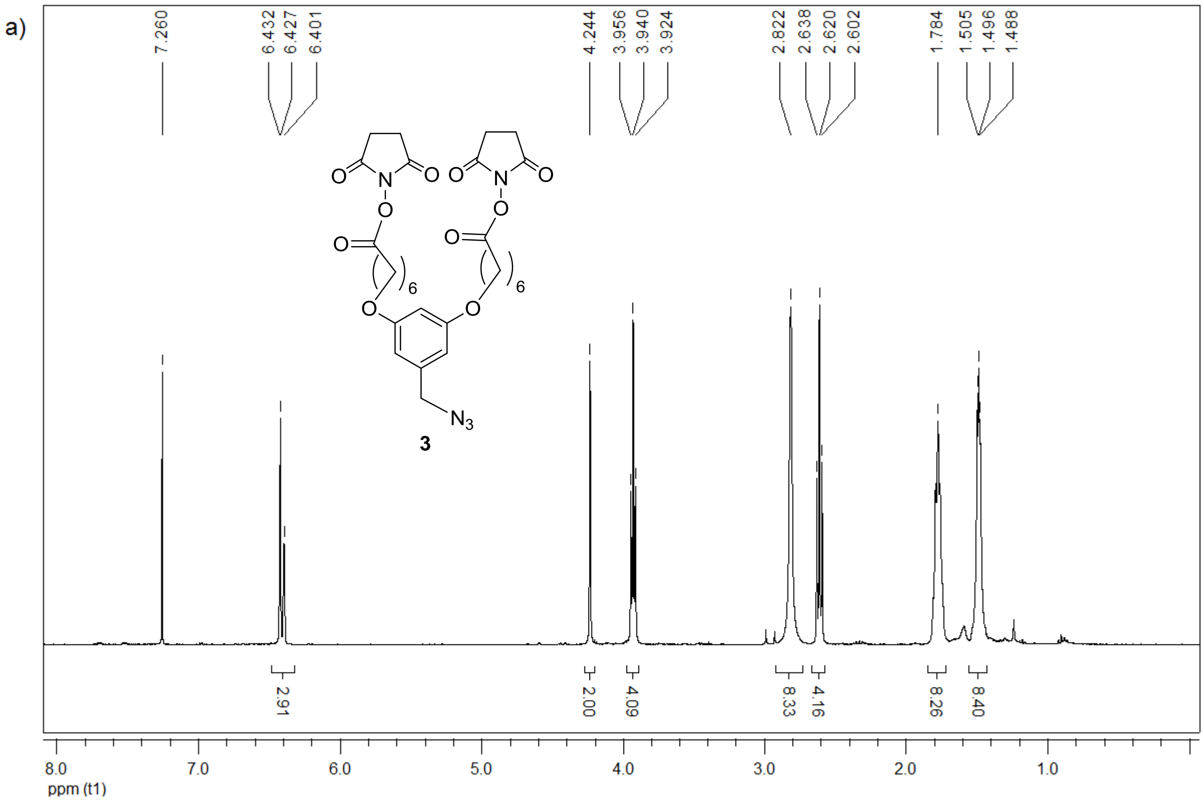

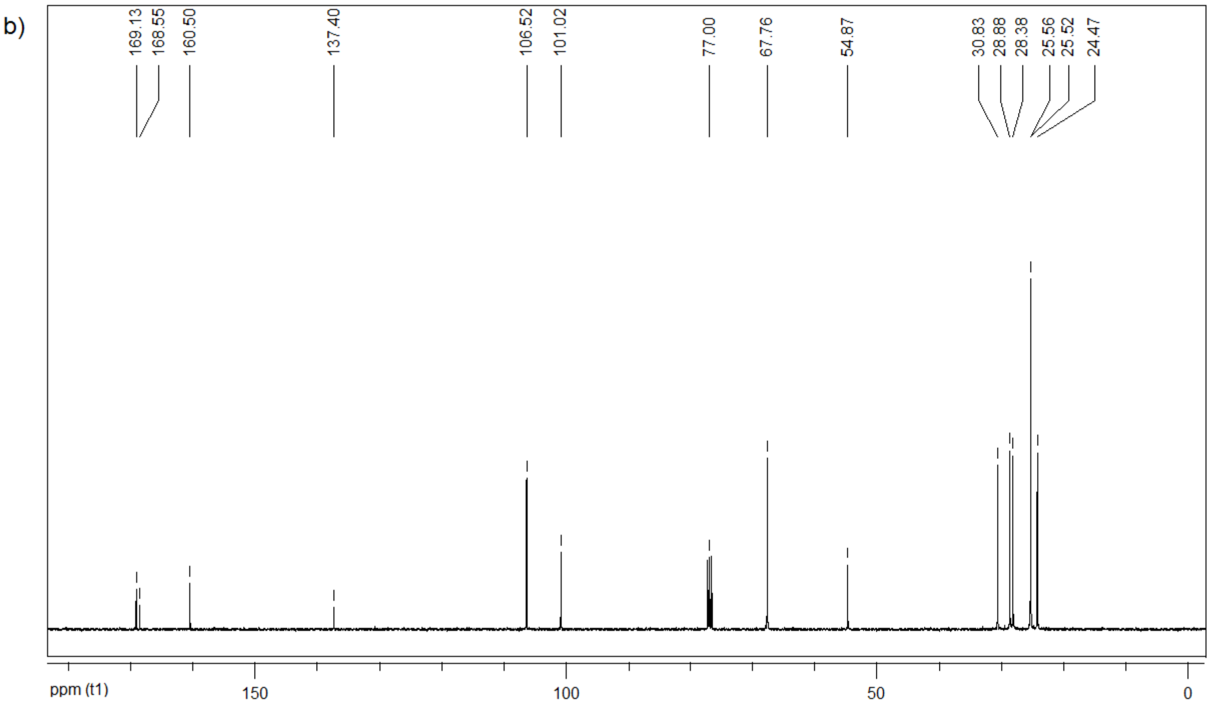

Figure S3. ^{1}H (a) and $^{13}C\{^{1}H\}$ (b) NMR spectra of compound **3** ($CDCl_3$).

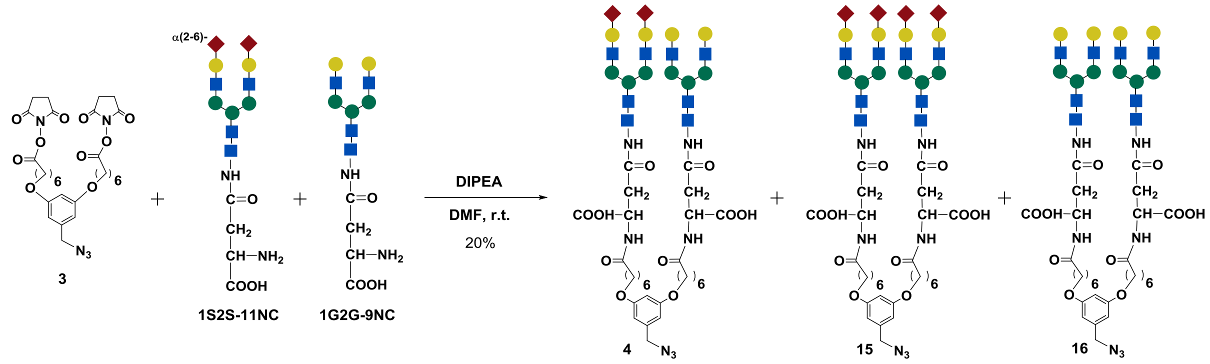

Scheme S2. Synthesis of bis-hetero-α(2,6)sialic acid-galactose terminated azide **4**.

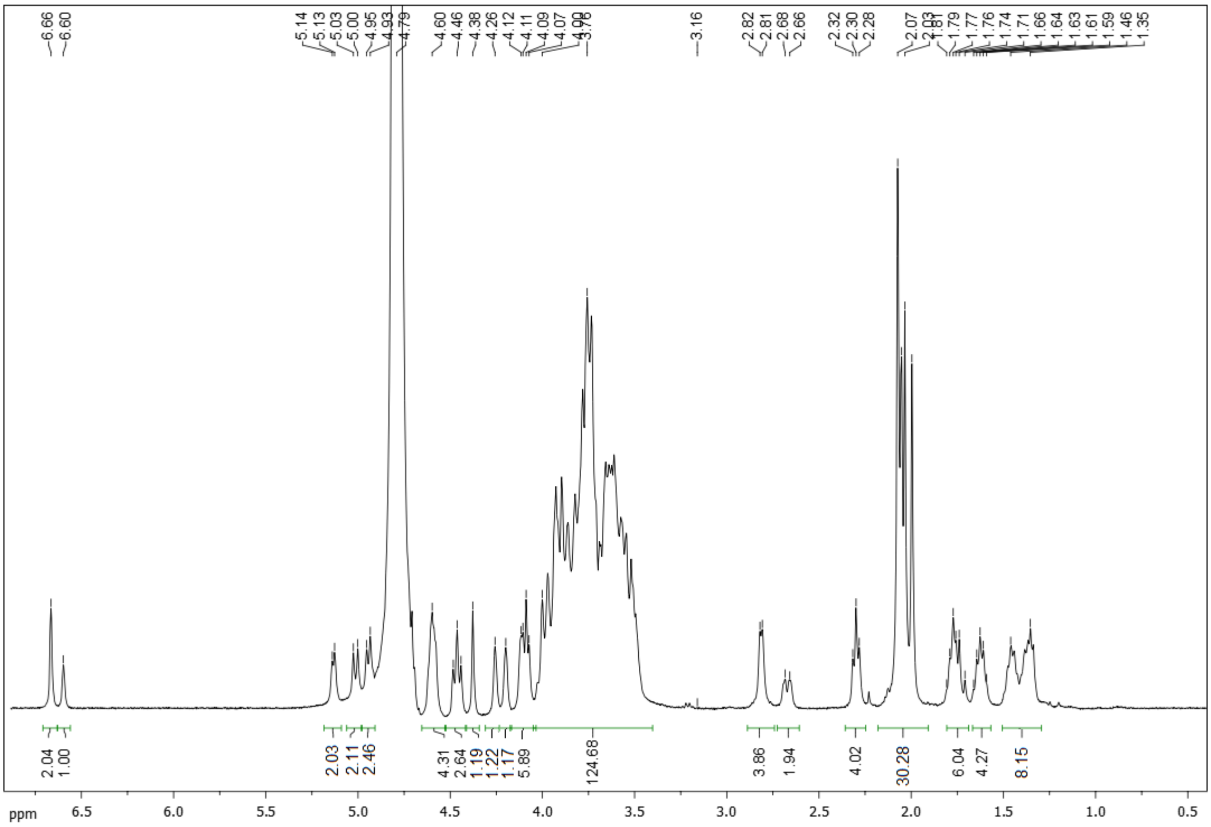

Figure S4. ^{1}H NMR spectrum of compound **4** (D_2O, 400 MHz).

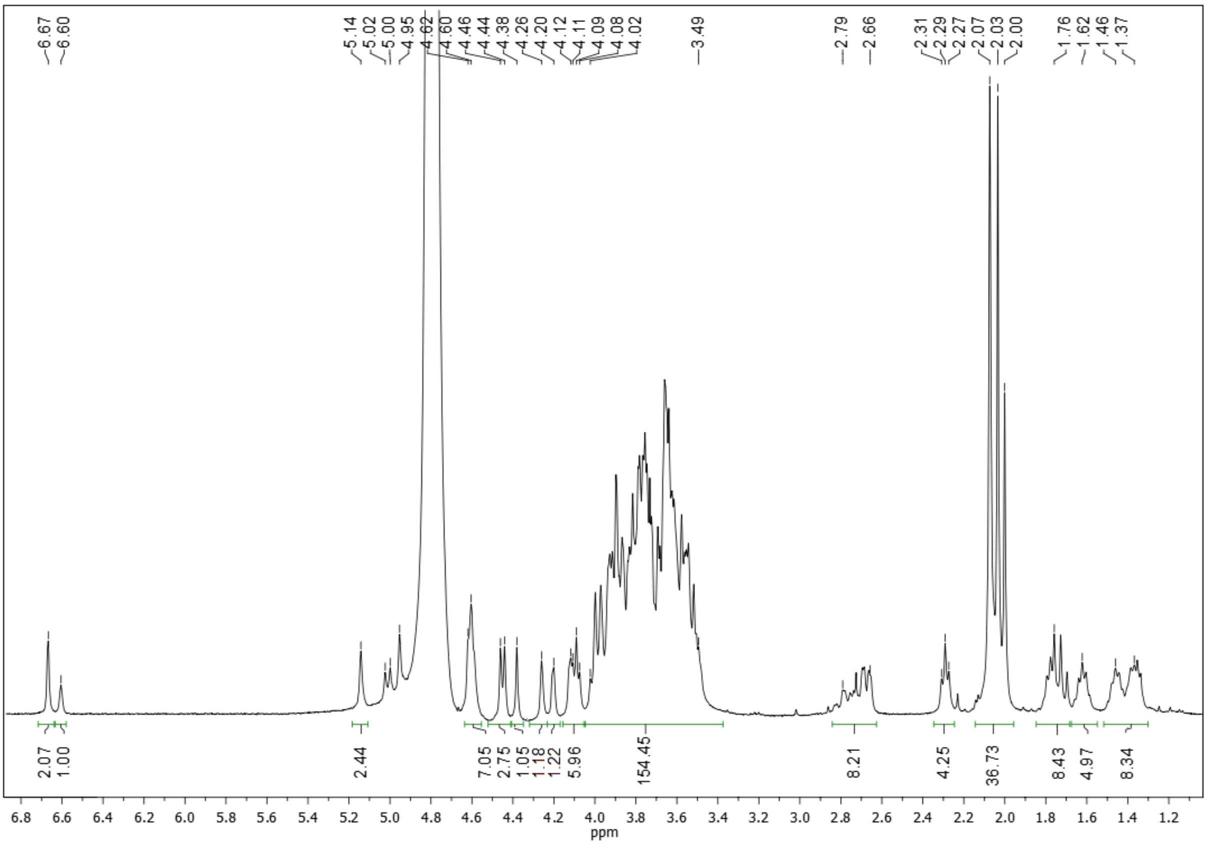

Figure S5. ^{1}H NMR spectrum of compound **15** (D_2O, 400 MHz).

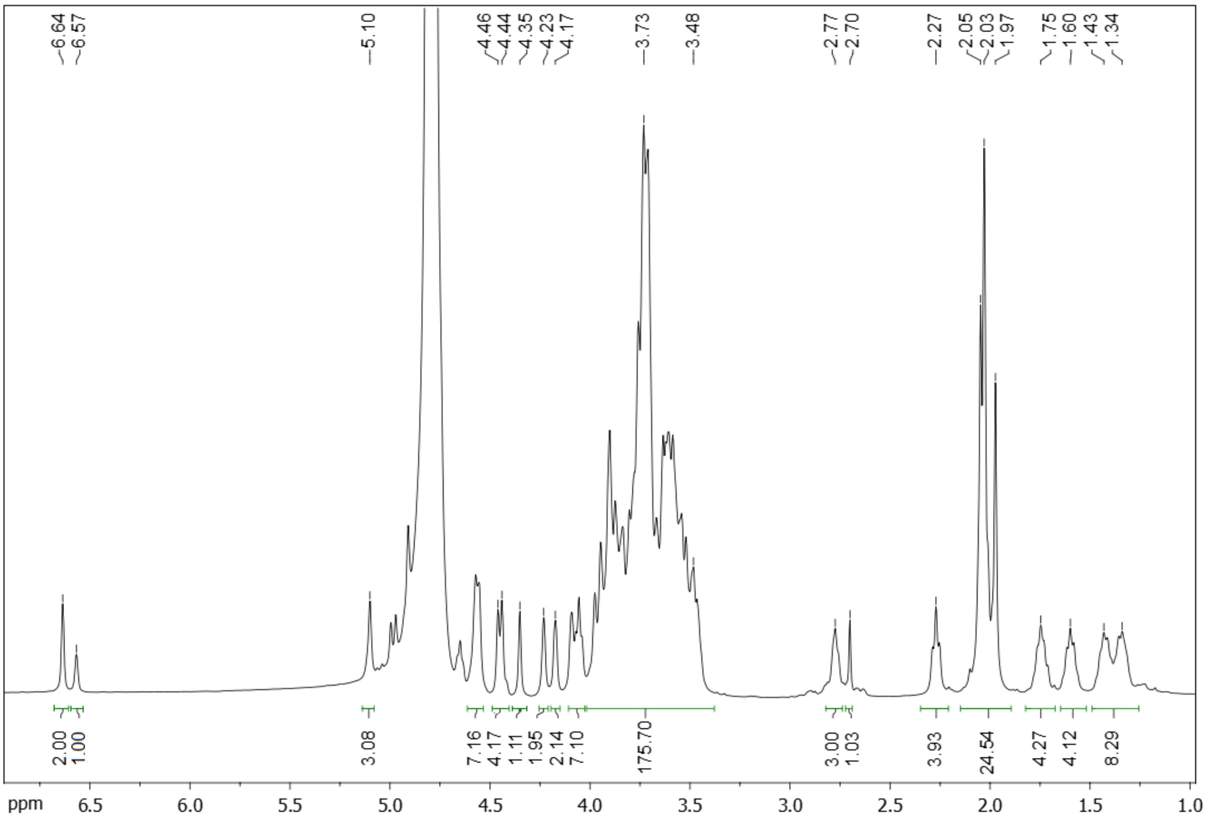

Figure S6. ^{1}H NMR spectrum of compound **16** (D_2O, 400 MHz).

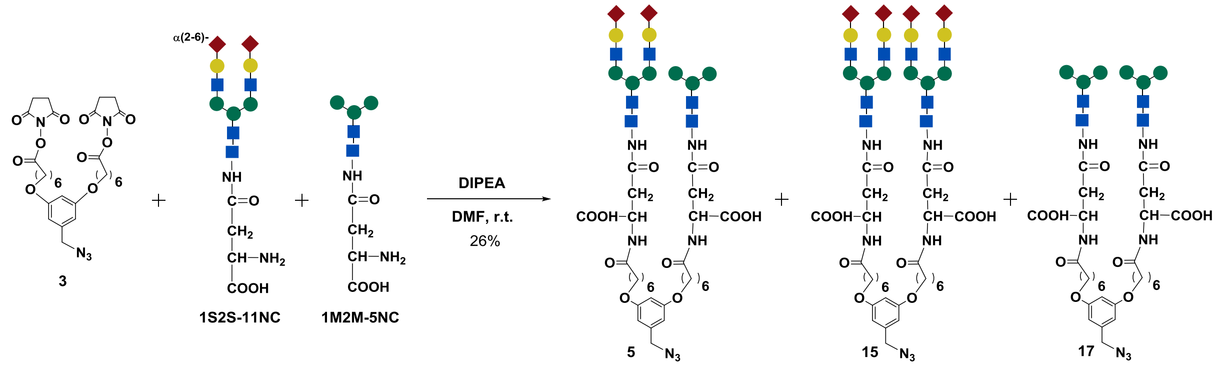

Scheme S3. Synthesis of bis-hetero-α(2,6)sialic acid-mannose terminated azide **5**.

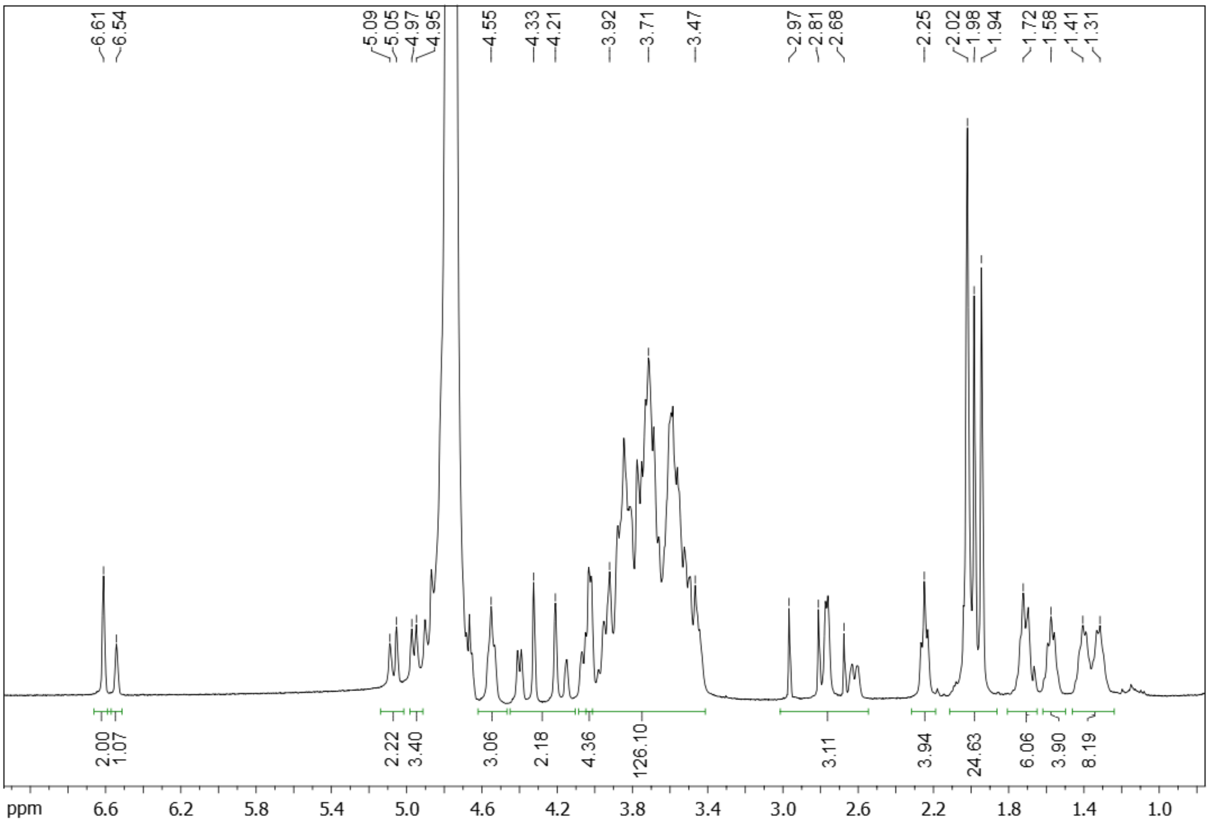

Figure S7. ^{1}H NMR spectrum of compound **5** (D_2O, 400 MHz).

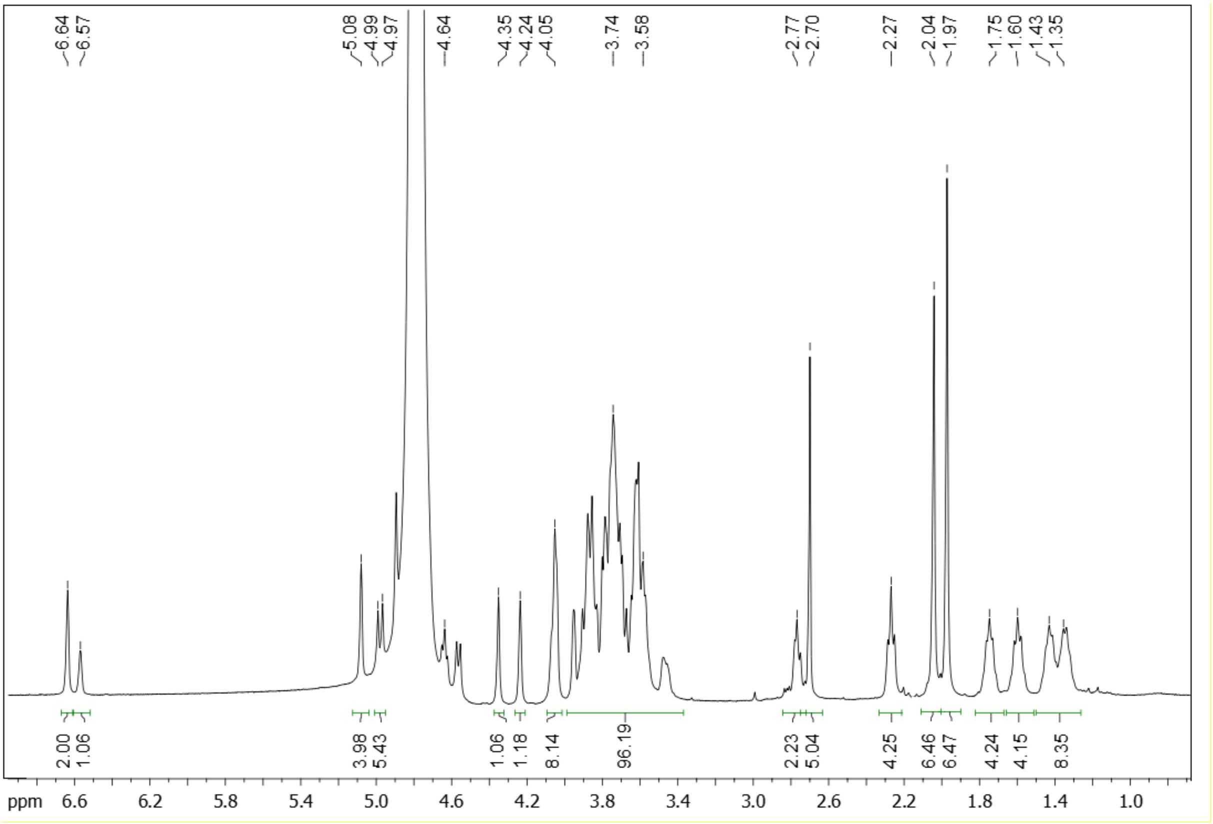

Figure S8. ^{1}H NMR spectrum of compound **17** (D_2O, 400 MHz).

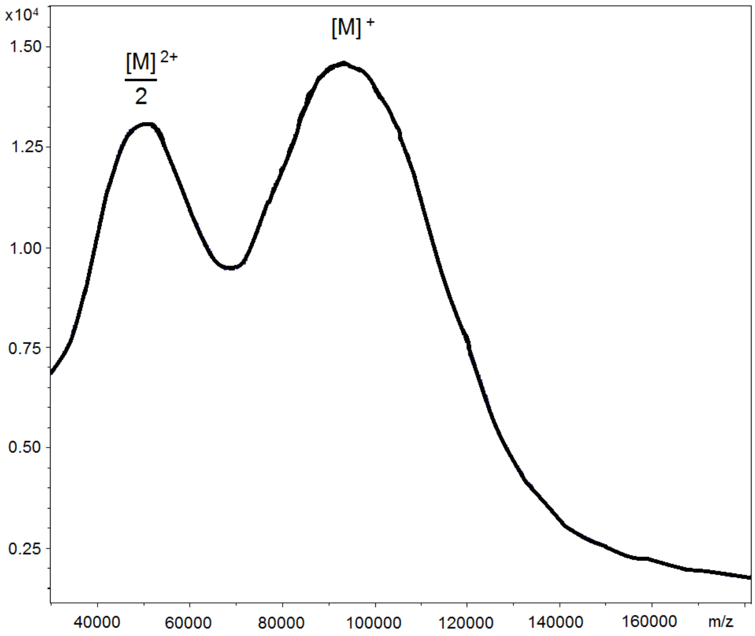

Figure S9. MALDI-TOF-MS of compound **8a** (positive mode).

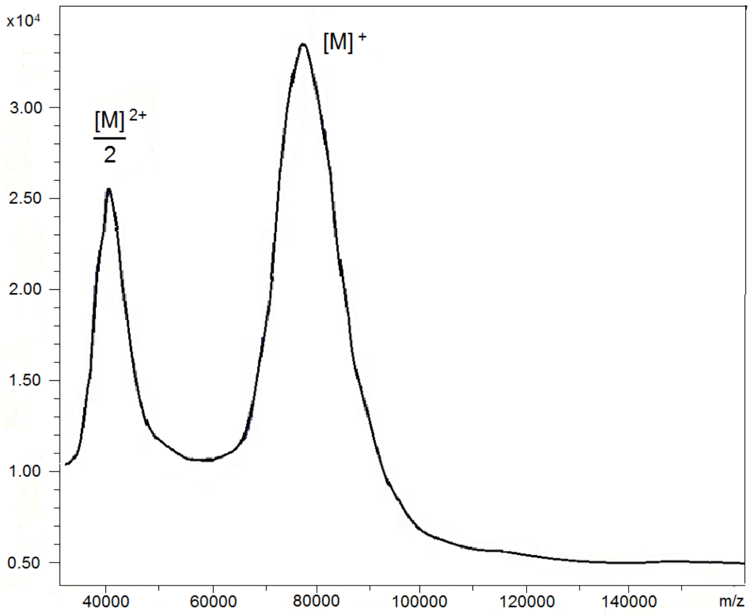

Figure S10. MALDI-TOF-MS of compound **8b** (positive mode).

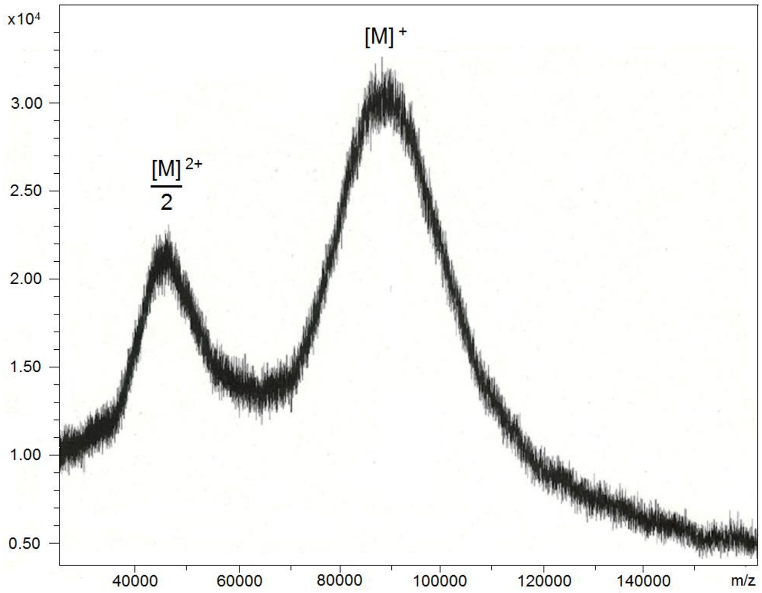

Figure S11. MALDI-TOF-MS of compound **9a** (positive mode).

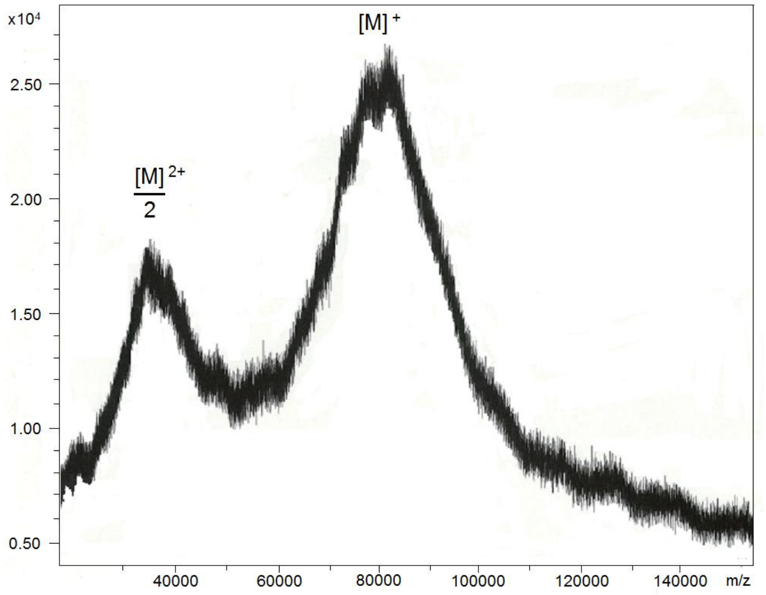

Figure S12. MALDI-TOF-MS of compound **9b** (positive mode).
